# A Mechanistic Understanding of the Different Factors Affecting Adaptation and Flexibility During Probabilistic Reversal Learning: A Neurocomputational Study on Patients with Parkinson’s Disease and Control Subjects

**DOI:** 10.1007/s10439-026-04038-4

**Published:** 2026-03-04

**Authors:** Giulia Piermaria, Silvana Pelle, Alessio Zizzi, Carola Cosentino, Susanna Mezzarobba, Giovanna Calandra-Buonaura, Luisa Sambati, Federica Scarpellini, Elisa Pelosin, Mauro Ursino

**Affiliations:** 1 Department of Electrical, Electronic, and Information Engineering “Guglielmo Marconi”, University of Bologna - Area di Campus Cesena, Via Dell’Università 50, 47521 Cesena, FC Italy; 2 Department of Neuroscience, Rehabilitation, Ophthalmology, Genetics and Maternal Child Health, University of Genoa, 16132 Genoa, Italy; 3 IRCCS Ospedale Policlinico San Martino, Genoa, Italy; 4 Department of Biomedical and NeuroMotor Sciences, Alma Mater Studiorum –University of Bologna, 40100 Bologna, Italy; 5 IRCCS, Istituto delle Scienze Neurologiche di Bologna, 40100 Bologna, Italy

**Keywords:** Reinforcement learning, Basal Ganglia, Parkinson’s disease, ON–OFF Levodopa medication, Reversal learning, Dopamine

## Abstract

**Purpose:**

Reinforcement learning with reversal is a paradigm frequently employed in neuropsychology to assess the adaptive capacity and flexibility in individuals in non-stationary environments. Each single behavior, however, depends on the superimposition of multiple factors, which are still not completely understood.

**Methods:**

We optimize a neurocomputational model of the Basal Ganglia to simulate the behavior of 18 patients with Parkinson’s disease (both ON and OFF medication) and 14 control subjects during a two-choice probabilistic reversal learning task (with 80–20% reward probabilities).

The individual behavior (in terms of the cumulative number of correct responses during a 40-trials direct phase and a 40-trial reversal phase) is reproduced by fitting a few model parameters for each individual, representing the tonic dopamine level, Hebbian learning, and the exploratory attitude (noise level).

**Results:**

Results show that very different responses can be explained quite well, ascribing them to the varying combination of the aforementioned individual factors. The tonic dopamine level is significantly different for patients in ON and OFF medication, while the other parameters were not statistically different. A regression analysis reveals that the value of the Hebbian learning rate is correlated with the subject's sensitivity to punishments. In contrast, the noise standard deviation is correlated with exploration, i.e., the tendency to modify the choice even after a reward.

**Conclusion:**

The results provide a mechanistic explanation of the various factors that affect adaptation and flexibility in reinforcement learning, representing a first step toward characterizing and understanding the diverse behaviors on an individual basis.

**Supplementary Information:**

The online version contains supplementary material available at https://doi.org/10.1007/s10439-026-04038-4.

## Introduction

Reinforcement learning (RL) is a fundamental adaptive process that enables a subject to learn from the environment, based on a previous history of rewards and punishments, without the need for an external teacher [1].

A pivotal role for RL in humans is played by the Basal Ganglia (BG), a cluster of subcortical nuclei involved in action selection under the control of dopamine (DA) tonic levels and phasic changes. The basic structure of the BG exhibits a Go pathway, which favors the occurrence of a given action, and a NoGo pathway, which inhibits that same action. Phasic peaks in dopamine levels, occurring after unexpected rewards, potentiate synapses in the Go portion of the Striatum, favoring a selected action. In contrast, phasic dips after unexpected punishments potentiate the NoGo portion, inhibiting the future selection of the present action. In patients with Parkinson’s disease (PD), insufficient DA production by the substantia nigra and the consequent DA level depletion can alter the previous subtle mechanism, causing well-known disturbances in motor responses (such as bradykinesia, tremor, freezing), a delay in action selection, and a progressive decline in cognitive capacities [2–4].

The RL paradigm is particularly challenged in non-stationary environments, such as those normally faced by humans. Suppose the environment is rapidly modifying responses. In that case, the BG must not only find the correct behavior to maximize rewards and minimize punishments, but also rapidly update its choices to adapt to new contingencies [5]. This behavioral flexibility is essential for survival and success. Often, flexibility is studied using a paradigm known as reversal learning, in which a previous association between actions and rewards (and between actions and punishments) is suddenly reversed without prior warning, compelling subjects to rapidly update their behavior [6].

Experimental and clinical data show that the capacity to perform direct and reversal learning varies widely among subjects and also differs between control subjects, PD patients on Levodopa medication, and PD patients off Levodopa medication. In particular, several results in the literature support this scenario, suggesting that ON-medicated PD patients exhibit better reward-based learning [7] but impaired punishment-based (reversal) learning compared to OFF-medicated patients [8–14]. In other PD subjects, however, these differences were not evident or provided contradictory results, and some studies failed to confirm the previous scenario [15, 16].

The reasons for large individual differences in responses during RL (even within the same group of participants) and the contradictory results found in PD patients are still not completely understood, and the mechanisms underlying them remain unclear. They may depend on a delicate balance among various factors that participate in the task and interact in complex ways. To explain the differences in RL between PD patients (especially during ON medication), some authors formulated the so-called hyperdose hypothesis [17–19]. Levodopa would induce an excess of DA concentration in a zone of the BG (especially the ventral one), which is only scarcely affected by pathological DA depletion, thus impairing certain cognitive functions – such as probabilistic reversal learning, motor sequence learning, or other cognitive tasks – driven by the surrounding dopamine-intact brain regions [19]. This effect depends on a complex balance between the Levodopa dose and the specific patient's disease stage. Unfortunately, differences between single PD subjects (early vs. late stage, sensitivity to levodopa) cannot be deduced from previous works. Other important factors, besides an altered tonic dopaminergic level and the related cholinergic effects, are as follows: i) the plasticity of striatal synapses during learning, allowing future maximization of rewards and minimization of punishments (*exploitation*); ii) the *exploration* of environmental novelties, generally represented as a random component of the individual choice, which contributes to the development of new selections [20].

To further investigate these aspects (exploration vs. exploitation, the effect of tonic DA levels, and direct vs. reversal learning) in this work, we leveraged a recent neurocomputational model of the basal ganglia [21–23] which can learn from reinforcement examples. In particular, we do not aspire to propose new mechanisms compared with previous studies, but to use an existing model previously validated on literature data [21], to characterize individual subjects (both healthy and PD). In particular, the aim is to ascribe the striking individual variability observed experimentally to a few factors with a clear behavioral and clinical impact. To this end, we compare model predictions with behavioral data obtained from a group of control subjects and PD patients at the early stage of the disease (disease duration < 5 years in both ON and OFF medication states) during a classic probabilistic reversal learning task. This task involves a two-choice learning procedure, where one choice is rewarded in 80% of cases, and the other choice is rewarded in the remaining 20% of cases, across 40 trials (direct phase). Then, these contingencies are suddenly reversed to test flexibility, and another group of 40 trials (reverse phase) is performed with inverted probabilities. Our model learns through a physiological Hebb rule proposed by the authors, driven by phasic DA changes following unexpected rewards and punishments [21]. Interestingly, with this model, we do not simply aim to simulate the average behavior of a group of subjects nor to reveal statistical differences between groups, as is done in previous works [11, 24], but to propose a new technique for characterizing individual participants using a small number of parameters with clear neurophysiological meaning and potential future clinical impact. Hence, we aspire to simulate each response pattern in both the direct and reversal phases and link this personal behavior to specific underlying mechanisms.

To this end, a fitting procedure was implemented by minimizing the squared difference between the cumulative number of correct responses, evaluated trial by trial, in the model and in the single participant. Fitting was achieved in each subject by assigning five essential parameters: the tonic DA level, the synapse learning rate, synapse saturation (upper and lower), and the noise standard deviation. The main assumption is that the combination of three essential mechanisms – dopaminergic influences, synapse learning (i.e., exploitation), and novelty exploration – can reproduce the richness of different individual responses discovered experimentally. We demonstrate that individual differences in RL can be explained quite well by assuming different combinations of the three contributions mentioned above. We found a significant correlation between each parameter and a specific behavioral index.

These results might contribute to a better understanding of the complex mechanisms that affect adaptive behavior (in the direct and reversal phases of learning) and provide indications of the effect of DA medication. In perspective, the model can provide indications for a customized rehabilitation protocol for PD patients and a framework to study other pathologies related to DA imbalances and BG dysfunction.

## Methods

### Study Population and Task Description

#### Participants

Patients with early PD and control subjects were recruited at the Movement Disorders Center, U.O.C. Clinica Neurologica Rete Metropolitana (NeuroMet), IRCCS Istituto delle Scienze Neurologiche di Bologna, and at the “Neurotunes” laboratory of the Department of Neuroscience, Rehabilitation, Ophthalmology, Genetic and Mathernal-Child Health (DINOGMI), of the University of Genoa. The study procedures (Clinical trial number: not applicable) were carried out in accordance with the Declaration of Helsinki (World Medical Association, 2013). Written informed consent to research and to publication of results was obtained from all participants before inclusion in the study project.

Common inclusion criteria were as follows: (i) age between 50 and 70 years old, (ii) no history of neurological disorders (except PD), (iii) absence of other medical conditions (e.g., orthopedic, psychiatric, cardiovascular diseases that could impact study results). Common exclusion criteria were as follows: (i) presence of cognitive impairment (Mini Mental State Examination, MMSE score < 26 [25]; Montreal Cognitive Assessment, MOCA score < 26 [26]; (ii) moderate/severe depression evaluated through Beck Depression Inventory [27]; (iii) presence of neurogenic orthostatic hypotension [28]; (iv) excessive daytime sleepiness (Epworth scale > 10) [29, 30]; (v) High risk of obstructive sleep apnea syndrome (Berlin Questionnaire) [31]; (vi) sleep disturbances that could impact study results (Parkinson’s Disease Sleep Scale, PDSS-2 score > 15) [32]; and (vii) denied oral and written informed consent to study participation.

In addition to these criteria, patients with PD were considered eligible if they had: (i) clinically established PD, according to international criteria [33]; (ii) disease duration ≤ 5 years; (iii) Hoehn & Yahr scale ≤ 2.5 [34]; (iv) absence of motor fluctuation (Movement Disorders Society Unified Parkinson's Disease Rating Scale, MDS-UPDRS Part IV score = 0) [35]; and (v) treated with a stable dose of immediate release formulation of L-Dopa/Carbidopa or L-Dopa/Berserazide for at least 3 months before the first evaluation. They were excluded in the presence of concurrent treatment with COMT inhibitors or MAO inhibitors. All subjects were unaware of the purpose and hypotheses of the study before participating, and they were informed about them after the experimental procedures were completed.

In the PD group, disease severity was measured with the MDS-UPDRS (part III) [35] and H&Y stage [34]. Daily Levodopa dose (LEDD) was collected.

For the control group, we collected data on age, sex, and education.

PD participants were asked to perform the task in the morning, either in an ON or in an OFF (before the first Levodopa dose) dopaminergic medication state.

OFF state is defined as the early morning condition of medication, following at least 12 h of withdrawal from short-acting Levodopa and 24 h from long-acting dopaminergic medications.

ON state is defined as the subjectively best motor condition, assessed about 60 minutes after intake of the usual morning dose of Levodopa.

A 12-h washout of any concomitant anti-Parkinsonian drugs was required before each session. Both experiments were performed approximately 7 to 14 days apart, in both the ON and OFF medication states. The order of the 2 experiments in ON or OFF conditions was randomly determined. The same procedure was applied for HS participants, except for those related to medication status.

#### Task Description

**Fig. 1:**
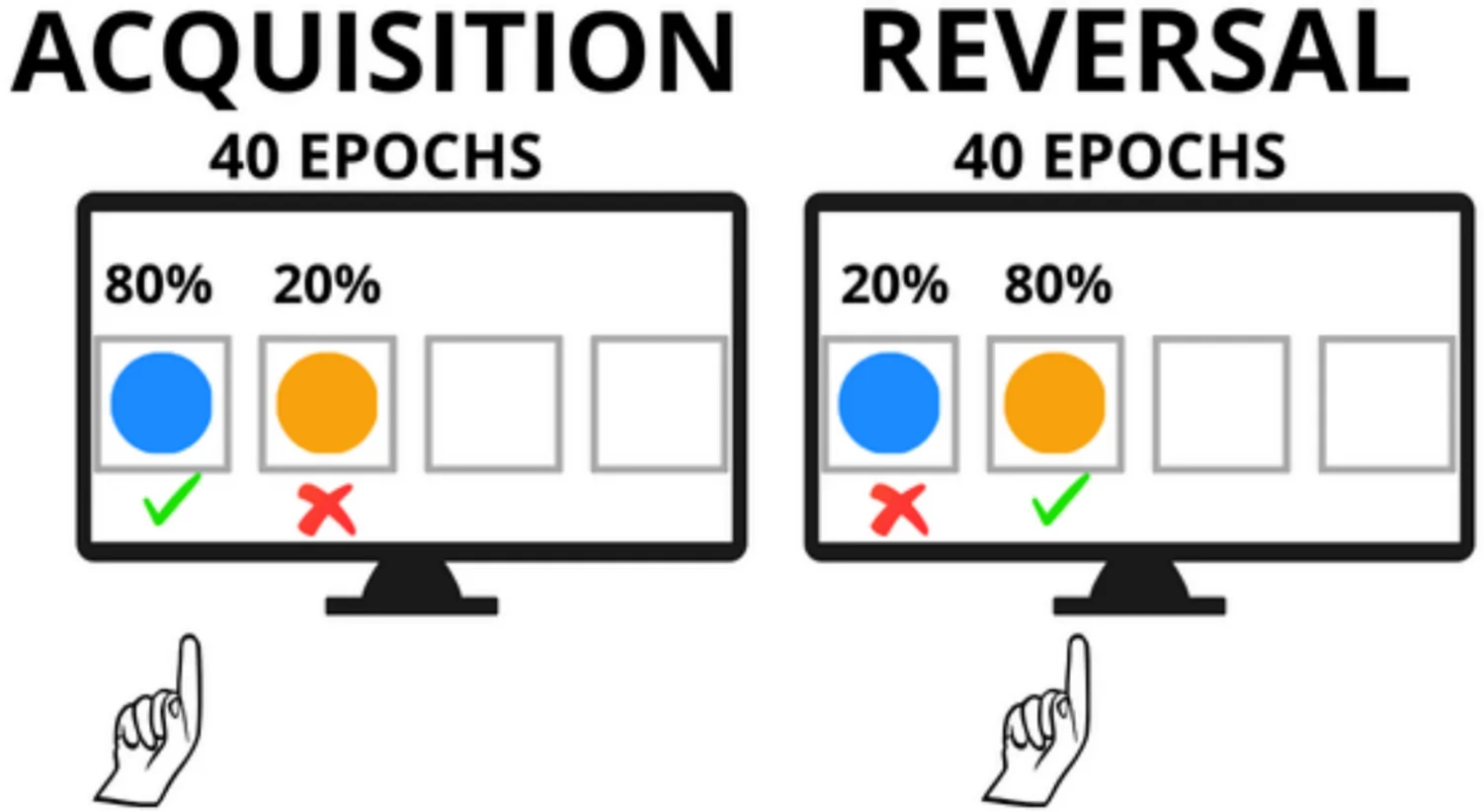
Schematic representation of the task. The task consists of two separate blocks: acquisition and reversal. On each trial, two colored shapes randomly appear inside four fixed squares on the screen and the participants should select one of them. One color (e.g., blue circle – 80% correct) is more frequently associated with correct feedback than the other (e.g., orange circle – 20% correct). During the acquisition block, participants must learn through trial and error which color is more likely to be correct and consistently select it. At an unpredictable point, the reversal block begins, during which the reward contingencies switch

We implemented a probabilistic RL task (Fig. 1). Participants were presented with two differently colored shapes (blue and orange) that were randomly placed in two of the four squares shown on the screen. They were required to discover which color would lead to a reward and avoid the punishment. As the task follows a probabilistic structure, selecting a specific color resulted in a reward 80% of the time, while in the remaining 20% of cases, it led to a punishment. Conversely, the other color followed the opposite probability pattern.

Specifically, participants were given the following instructions:

“On the screen, you will see four squares within which two shapes of different colors will appear. You will need to choose one of the two colors, and the computer will indicate whether your choice was correct or incorrect. Each color will sometimes be correct and sometimes wrong, but one of the colors will tend to be correct more often than the other. You will need to determine which color is typically correct and select that color each time. Be sure to stick to that one, even if it is occasionally wrong. Be careful because at some point, the usually correct color may change, and as a result, the other color will become the usually correct color. In that case, you will have to choose that color.”

In this experiment, reversal occurred only once per participant, maintaining a clear distinction between the acquisition and reversal phases. The acquisition phase consists of 40 epochs. The first participant's choice was always assumed to be the correct response (and subsequently rewarded in 80% of cases) and was initially rewarded. This choice was made to ensure that all participants would start with the same condition. At the start of the following reversal phase, which also consisted of 40 epochs, reward and punishment probabilities were oppositely associated with the colors. To evaluate the participant’s performance and compare it with the model’s results, we computed two *cumulative functions* independently during the direct and reversal phases. This function sums up the number of correct responses up to the present trial. Hence, it is a monotonic function that starts at zero and could reach a maximum value of 40. Moreover, we computed a *final score*, which is the number of correct responses achieved after 40 trials; hence, the final score equals the last value of the cumulative function.

It is important to keep in mind that a correct response implies 80% rewards and 20% punishments, and that the response is inverted from the direct to the reversal phase.

### Qualitative Model Description

The model represents the human BG based on previous works [21–23]. Figure S1 in the Supplementary Materials provides a schematic representation.

The network presents several units, each simulating a group of neurons with similar functions. Each unit's dynamics can be described by a first-order low-pass filter that mimics the integrative properties of the synapse and membrane and by a sigmoidal relationship for the output activity, indicating upper and lower saturation values.

The model includes both a sensory representation, which codes the input stimulus, and a motor representation in the cortex, which encodes possible actions. In addition, the model includes the striatum, further subdivided into Go and NoGo pathways, the Globus Pallidus pars externa (Gpe), the Globus Pallidus pars interna (Gpi), and the Thalamus (T). Every units in these structures have a 1-to-1 relationship with neurons in the motor cortex, representing the possible actions. Conversely, the cholinergic area (ChI) and the subthalamic nucleus (STN) are modeled with a single unit, which affects the overall structure.

In the current model version, only two actions are included (as shown in Figure S1), corresponding to the two possible choices in our task.

To simulate exploration, Gaussian noise with a zero-mean value and a specified standard deviation (SD), denoted as parameter *n*, has been added to the two neurons in the motor cortex, thereby modifying the Winner-Takes-All (WTA) dynamics.

The neural units are connected to realize the three main pathways responsible for BG functioning: direct (via Go neurons), indirect (via NoGo neurons), and hyperdirect (via the STN). The result of these pathways modulates the inhibition of the thalamus from Gpi, either permitting or blocking a coded response by motor neurons.

At basal steady state, the network exhibits inhibited activity in the Cortex, Striatum, and Thalamus, with basal activity in the Gpi and Gpe, following physiological data [36]. However, in the presence of sufficient stimulation, the Motor Cortex can select an action through a WTA mechanism. During this process, the direct and indirect pathways operate in parallel; the direct pathway facilitates the response by directly inhibiting the Gpi, while the indirect pathway blocks the response by inhibiting the Gpe. The hyperdirect pathway helps resolve intense conflict among cortical units by blocking responses and providing additional time for the cortex to select a winning neuron. In detail, the STN receives a conflict level as input, and it sends excitation to the Gpi, providing additional thalamic inhibition.

The Hebbian rule is used to train the network in response to rewards and/or punishments, modifying synaptic weights that enter the Striatum based on the activity of the Go and NoGo neurons:

1 $$\Delta w_{ij}^{AB} = \gamma \left( {y_{j}^{B} - \vartheta^{PRE} } \right)\left( {y_{i}^{A} - \vartheta^{POST} } \right)$$

where $\Delta {w}_{ij}^{AB}$ is the variation of the synapse between the pre-synaptic neuron j in layer B (B = S or C) and the post-synaptic neuron i, in layer A (A = G or N), *y* represents neuronal activity (normalized between 0 and 1), ${\vartheta }^{PRE}$ and ${\vartheta }^{POST}$ are the thresholds for the pre-synaptic and post-synaptic activity, and γ is the learning factor.

Moreover, we assume that the synapse values cannot increase above an upper saturation value, nor decrease below a lower saturation value.

Since rule (1) is extremely important to our estimation process, it warrants justification. First, when both the pre-synaptic and post-synaptic neurons are above threshold, the rule involves synaptic potentiation. Second, when one neuron is above threshold, and the other is below threshold, the rule involves depotentiation (either heterosynaptic or homosynaptic). All these changes are well documented in the literature. However, the rule also assumes that when both neurons are below threshold, a synaptic potentiation occurs, a result that does not seem physiological. However, as already discussed in our previous work [21], this rule can be justified physiologically, assuming that synapses from the cortex to the striatum are not only excitatory, but also involve an inhibitory branch. As shown in Fig. S2 in the Supplementary Materials, the total “apparent” synapse linking the cortex to the striatum may result from a parallel “push-pull” arrangement between an excitatory pathway and a disynaptic inhibitory pathway. This hypothesis assumes the presence of inhibitory interneurons in the striatum, which are normally moderately active. Consequently, when both cortical and striatal projection neurons are inhibited (hence below threshold), the excitatory synapse linking the cortical neuron to the striatal inhibitory interneuron experiences heterosynaptic depotentiation. This results in an increase in the total synapse strength. Experimental data supporting the presence of inhibitory interneurons in the striatum, and their involvement in learning, are discussed in the final section.

Synaptic changes in Eq. (1) are modulated by phasic dopamine changes, which affect the post-synaptic neuron activity: unexpected rewards result in long-term potentiation for the winner neuron in the Go pathway and long-term depression for the winner neuron in the NoGo pathway; unexpected punishments result in potentiation of the winner NoGo and depotentiation of the winner Go. However, these changes are strongly influenced by the tonic DA prior to the receipt of rewards and punishments.

More in detail, the phasic DA signal is shaped by the reward expectation: a high effect (peak or dip) of phasic DA follows an unexpected outcome (reward or punishment). In contrast, a negligible effect occurs when an expected outcome is achieved. The equations are

2 $$\Delta D_{reward} = \left[ {2*\left( {1 - r_{expected} } \right)} \right]^{m}  D_{p}$$

3 $$\Delta D_{punishment}=-0.5 [2*r_{expected}]^{m}D_{p}$$

where $\Delta {D}_{reward}$, $\Delta {D}_{punishment}$ are the phasic DA peak following a reward and the phasic DA dip (hence negative) following a punishment, respectively. *m* is a parameter equal to 2.5, empirically assigned [22], *D*_*p*_ is a constant parameter setting the strength of the phasic DA changes, and *r*_*expected*_ is the value of the winning go neuron at the instant in which a reward/punishment is given, signaling a reward expectation.

According to Eq. (2), an unexpected reward (*r*_*expected*_* ~* 0.5) produces a high phasic dopamine peak ($\Delta {D}_{reward}={D}_{p}$), whereas, according to Eq. (3), an unexpected punishment (*r*_*expected*_ ~ 1,0, signifying a high expected reward) produces a large dopamine dip.

To simulate the present task, we hypothesize that the network starts from a completely naïve state, wherein all synapses initially have equivalent values, and none of the two actions is preferred. To further clarify, the synapses from the sensory cortex neurons to the Go and NoGo neurons, as well as those from the motor cortex neurons to the Go and NoGo neurons, are set to 0.5, ensuring an unbiased starting point. Moreover, as in the experimental set-up, the first random choice is always assumed to be the correct one (hence, subsequently associated with an 80% reward probability), regardless of the winning motor neuron that was initially chosen and initially rewarded.

Exposure to the stimulus is long enough to allow the network to select a winner and subsequently reach a new steady-state condition. Only once the final steady state is reached, a punishment or a reward is given, and synaptic training is performed using the Hebbian paradigm. During all trials (40 in the direct phase and 40 in the reward phase), the network was exposed to the same sequence of correct responses (80% of one type and 20% of the other type) as the human participant, ensuring consistency in stimulus presentation.

In the following, five model parameters will be estimated for each participant by minimizing a cost function. These parameters are the tonic DA level, the learning factor γ, the SD of noise, and the upper and lower saturation levels for the striatal synapses. All other parameters have the same value as in Ursino et al. [21].

### Pattern Search Algorithm

As specified above, five parameters in the model (the tonic DA level, the learning factor γ, the SD of noise, and the upper and lower saturation for the striatal synapses) were individually assigned for each session by minimizing a cost function of the difference between the cumulative functions in the participant and the model. Specifically, the model was provided with the same sequence of 80–20% correct responses used for the participant in the direct and reversal phases. Ideally, it should produce responses that align with those of the subject. The cost function computes the sum of squared errors (SSE) defined as follows:

4 $$SSE\left( \theta \right) = \mathop \sum \limits_{{i_{acquisition} = 1}}^{40} \left( {x_{p,i} - x_{m,i} \left( \theta \right)} \right)^{2} + \mathop \sum \limits_{{i_{reversal} = 1}}^{40} \left( {y_{p,i} - y_{m,i} \left( \theta \right)} \right)^{2}$$

where ${x}_{p,i}$ represents the value of the cumulative function evaluated on the participant after the i-th trial of the acquisition stage, ${x}_{m,i}(\theta )$ denotes the corresponding cumulative function computed by the model, which is, of course, a function of the estimated parameters *θ*. Similarly, ${y}_{p,i}$ and ${y}_{m,i}(\theta )$ are the cumulative functions evaluated during the reversal phase.

To determine the parameter values that best replicate the subject's cumulative function, we performed an optimization procedure using the pattern search algorithm [37, 38], which is particularly well suited for nonlinear objective functions, as in the case of this study. The pattern search optimization was implemented using the MATLAB Optimization Toolbox (MathWorks Inc., Natick, MA), specifically the *patternsearch function.* The optimization was performed in a bounded parameter space, preventing the algorithm from exploring unrealistic regions. This function is an optimization method that iteratively evaluates the output of a cost function using spanning sets, formed by the canonical basis vectors and their negatives, ensuring convergence toward a local minimum of the objective function without requiring gradient information. At each iteration, the algorithm evaluated the cost function at neighboring points around the current estimate and accepted a new point only if it reduced the sum of squared errors.

Initial parameter values were selected within physiologically plausible ranges and constrained by lower and upper bounds to ensure biologically plausible solutions.

Once a final least squares estimate was obtained using the minimization procedure, an approximate evaluation of the covariance matrix *V*(*θ*) of the parameter estimates was computed by assuming [39]

5 $$V\left( {\hat{\theta }} \right) = \sigma^{2} \left[ {S^{T} S^{ - 1} } \right]$$

where ${\sigma }^{2}$ denotes the variance of the measurement noise (assumed equal for all the data points) and *S* is the *NxP* sensitivity matrix (where *N* is the number of data points, equal to 80 in the present cost function, and *P* is the number of parameters to be estimated, equal to five) evaluated at the final parameter estimates $\theta =\widehat{\theta }$.

The sensitivity matrix was computed using a forward difference approximation for the time derivatives. As usual, ${\sigma }^{2}$ was estimated through the following equation

6 $$\sigma^{2} = \frac{{SSE\left( {\hat{\theta }} \right)}}{N - P}$$

Finally, from the knowledge of the covariance matrix, we evaluated the standard deviation of the pth parameter estimate (say *SD*_*p*_) as follows

7 $$SD_{p} = \sqrt {v_{pp} \left( {\hat{\theta }} \right)}$$

where ${v}_{pp}$ is the pth element of the principal diagonal of the covariance matrix.

### Multivariate Regression Between the Estimated Parameters and the Final Score

To evaluate the impact of model parameters on learning, we computed a multiple regression between the final scores (i.e., the values of the cumulative functions after 40 trials, *x*_*m,40*_ and *y*_*m,40*_ in Eq. 4) and three estimated parameters in the model: the tonic dopamine, the learning rate, and noise SD. As shown in the section results, only the dopamine level is significantly different between the PD ON, PD OFF, and control subjects, whereas the other parameters do not exhibit significant differences between the three groups; only for tonic dopamine, we used a piecewise relationship between the predictor variables and the response variable, segmented into three intervals. Moreover, different regressions were computed during acquisition and reversal. We have

$$Final score_{acq} = \left\{ {\begin{array}{*{20}c} {b_{1} + b_{4} *DA + b_{5} *\gamma + b_{6} *n } \\ {b_{2} + b_{5} *\gamma + b_{6} *n } \\ {b_{3} + b_{7} *DA + b_{5} *\gamma + b_{6} *n} \\ \end{array} } \right. \begin{array}{*{20}c} {DA < 0.9 \left( {PD OFF} \right)} \\ {0.9 \le DA < 1.1 \left( {control} \right)} \\ {DA \ge 1.1 \left( {PD ON} \right)} \\ \end{array}$$

$$Final score_{rev} = \left\{ {\begin{array}{*{20}c} {c_{1} + c_{4} *DA + c_{5} *\gamma + c_{6} *n } \\ {c_{2} + c_{5} *\gamma + c_{6} *n } \\ {c_{3} + c_{7} *DA + c_{5} *\gamma + c_{6} *n} \\ \end{array} } \right. \begin{array}{*{20}c} {DA < 0.9} \\ {0.9 \le DA < 1.1} \\ {DA \ge 1.1} \\ \end{array}$$

where DA is the tonic dopamine level estimated in the participant, *γ* is the estimated learning factor (eq. 1), *n* is the standard deviation of noise, and the *Final score* is the final score of that participant (equal to the last value of the cumulative function).

Please note that parameter DA is not used in the regression line for the mid-interval (control) since it exhibits only minor changes in control subjects.

Parameters in the regression curve were estimated using MATLAB’s function “*fitnlm,*” which minimizes the sum of squared residuals between the model predictions and the observed values via the Levenberg–Marquardt nonlinear least squares algorithm [40, 41].

### Behavioral Indices

At the end of the analysis, we examined the potential relationship between the model’s estimated parameters and some behavioral indices computed on the participants. In the literature, noise is often associated with the level of exploration, while the learning factor is linked to the velocity of changing the preferred choice [42–44]. Based on these ideas, we define some behavioral indices that could serve as proxies for exploration and flexibility.

To quantify exploitation, we defined an index named the punishment sensitivity as:

$${\text{Punishment sensitivity}} = \frac{{N_{{\mathrm{switch,punish}}} }}{{N_{{{\mathrm{punish}}}} }}$$

where ${N}_{switch,punish}$ represents the total number of times the subject switches their response after receiving a punishment and ${N}_{punish}$ denotes the total number of punishments received by the subject. Essentially, the index quantifies how frequently a subject alters his/her response following negative feedback. A lower punishment sensitivity suggests a stronger exploitation of previous learning, potentially indicating reliance on an established strategy. In contrast, a higher punishment sensitivity denotes greater behavioral flexibility in response to punishment, reflecting an adaptive learning process.

Moreover, we defined the exploration factor as:

$${\mathrm{Exploration}} = \frac{{N_{{\mathrm{switch,reward}}} }}{{N_{{{\mathrm{reward}}}} }}$$

where$N_{{{\mathrm{switch,}}\,{\mathrm{reward}}}}$ is the total number of times the subject switches his/her response after receiving a reward and $N_{{{\mathrm{reward}}}}$ denotes the total number of rewards received by the participants. This index measures how often a subject changes response despite having previously received positive feedback. The name of the index is self-explanatory: A high value indicates greater exploratory behavior, as the subject continues to switch responses even when rewarded. Conversely, a lower value indicates that the subject is more likely to stick with a response that has yielded positive outcomes, suggesting a more deterministic or exploitative strategy.

Again, we found a relationship between some of the model-estimated parameters and the behavioral indices (see Results). In particular, a nonlinear parabolic relationship could be observed between the punishment sensitivity and the learning factor:

$${\text{Punishment sensitivity}} = b_{1} + b_{2} \gamma + b_{3} \gamma^{2}$$

Conversely, we observed a linear regression between the exploration and the standard deviation of noise:

$${\mathrm{Exploration}} = c_{1} + c_{2} n$$

The regression parameters were obtained using MATLAB’s function “*fitnlm”* for minimization.

## Results

### Statistical Results for Patients and Control Subjects

Descriptive statistics for demographic and clinical characteristics, as well as behavioral outcome measures of all participants, are reported below.

Eighteen individuals with PD (six females, age range 46–67, mean age 58.8 ± 6.01 years) and fourteen healthy adults (nine females, age range 52–76, mean age 63.9 ± 6.60 years) met the inclusion criteria and were accepted to participate in this study. No statistically significant differences were found between male and female patients in any of the scores.

**Fig. 2:**
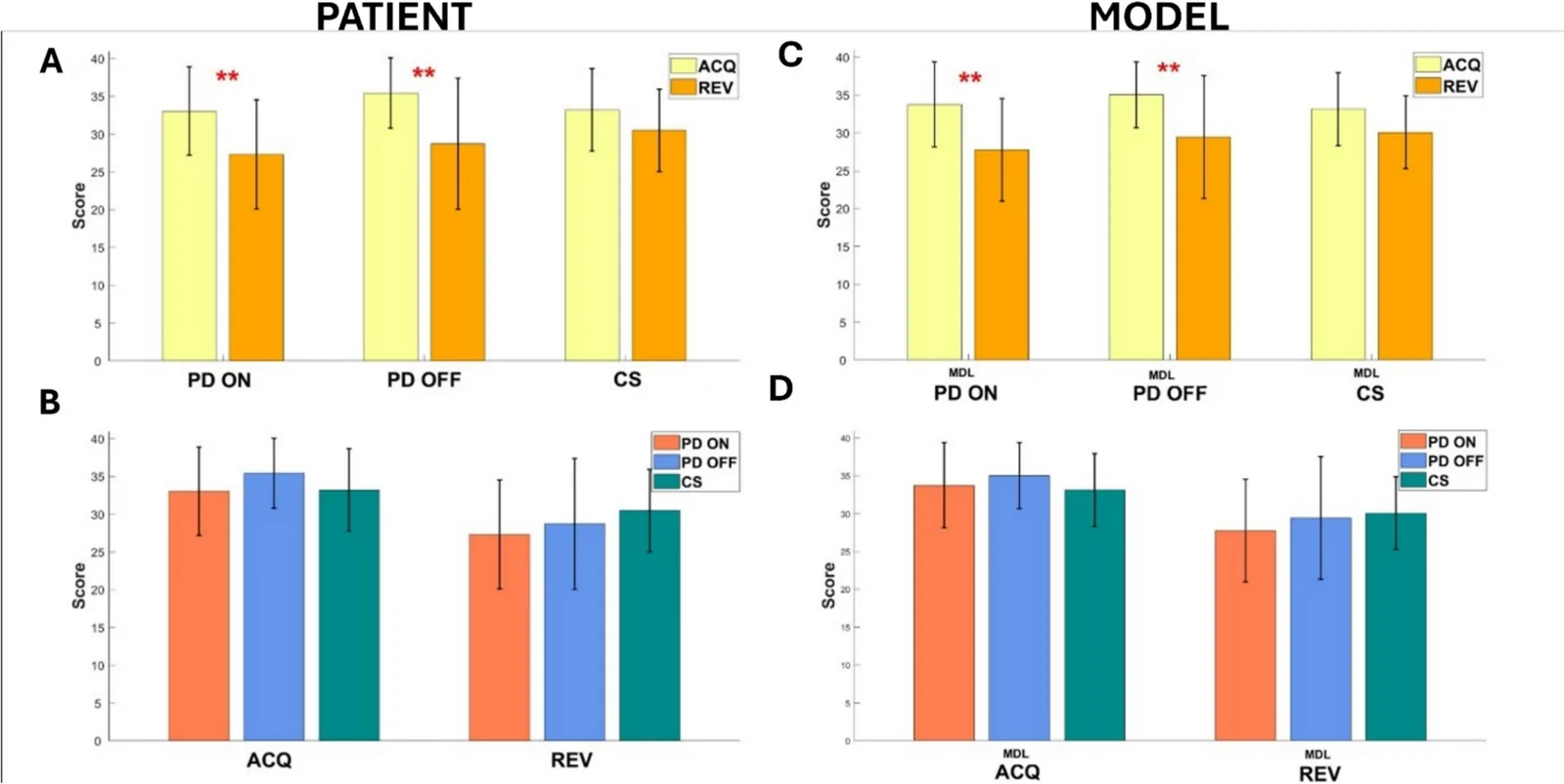
Statistical results on subjects’ data (left panels): **A** comparison of the final scores obtained during the acquisition and reversal phases in patients with Parkinson’s disease (PD ON, PD OFF, and CS). Panel **A** shows that the difference between the acquisition and reversal phases is significant for the PD ON and PD OFF conditions (0.001 ≤ *p*-value ≤ 0.01), but not significant for the control group. These results highlight that patients with Parkinson’s disease are more likely to show differences in their scores between the two phases. Panel **B** shows that there is no significant difference, as determined with pairwise Mann–Whitney U tests, between the three conditions (PD ON, PD OFF, and CS) in either the acquisition or reversal phases. Statistical results (right panels): Comparison of final scores obtained during the acquisition and reversal phases in the model representation of Parkinson’s patients (PD ON, PD OFF, and CS). Both panels (**C** and **D**) present statistical results for the final scores reached by the model, using the parameter inputs from Tables S1 and S2 in the Supplementary Materials. Panel **C** shows that the difference between the acquisition and reversal phases is significant for the model representation of PD ON and PD OFF conditions (**: 0.001 ≤ *p* ≤ 0.01), but not for the simulation of the control group. These results demonstrate that the model accurately captures the behavioral differences observed in patients with Parkinson’s disease. Panel **D** highlights that there is no significant difference between the three simulated conditions (PD ON, PD OFF, and CS) in either the acquisition or reversal phases, consistent with the patient data.

First, we statistically compared the final score (i.e., the number of correct responses at the end of the 40-trial session) between the acquisition and reversal phases within each group (see Fig. 2a) and between groups (ON, OFF, and control) (Fig. 2b). Panel A presents the results of the statistical analysis using the Wilcoxon method with Bonferroni correction for PD ON, PD OFF, and control subjects (CS). The comparison between the acquisition (yellow bar) and reversal (orange bar) phases reveals a significant difference in scores for patients in both conditions, as indicated by * in red. In contrast, the difference is not significantly different in control subjects. Furthermore, comparing patients (ON and OFF medication) with control subjects (Fig. 2b) shows no significant difference between groups in either the acquisition or reversal phases, although a decrease in the reversal score (but not statistically significant) is evident in the PD ON group.

### Parameter Estimates in Patients with Parkinson’s Disease and Control Subjects

The parameter estimates, along with their SD (computed as described in the Method section), obtained from the 18 patients (both ON and OFF medication) and the 14 control subjects, are presented in Tables S1 and S2 of the Supplementary Materials. The main results can be summarized as follows:

(i) All control subjects can be simulated satisfactorily using a tonic dopamine level close to the basal value (DA = 1 in the model). All patients in the OFF condition can be simulated with a lower DA value (range: 0.48–0.9), whereas the same patients in the ON condition require a higher DA value (range: 1.2–2.6). These results confirm the model’s ability to accurately mimic different dopamine conditions. The use of a higher-than-normal tonic dopamine in the ON condition conforms with the overdose hypothesis (see Discussion).
(ii) The ranges of values for the learning factor ([1.0–3.30] for the ON cases, [0.55–3.91] for the OFF cases, and [0.80–3.86] for the control group) are quite similar, without statistical differences. This indicates that learning capacity is not clearly affected by the pathological condition or by dopamine levels. However, large differences in this parameter can be observed among subjects, indicating that some have a lower and others have a higher learning capacity. Interestingly, most patients with a high estimated learning factor (greater than 1.75) exhibit high values both during ON and OFF medication states. Similarly, patients with a lower learning factor (<1.60) exhibit low values in both conditions. This supports the reliability of the estimates. The only exceptions are patients 4 and 11, who exhibit large differences between the two conditions. In the following section, this parameter will be linked to the subject’s behavioral index, “punishment sensitivity.”
(iii) Also, as to the noise level, large differences can be found among individuals (range [0.02–0.36] in the ON cases, [0.03–0.277] in the OFF cases, and [0.02–0.10] in the control group, but without statistical differences). Once again, in most cases, rather similar values can be used in both tests for the same patient. Eight patients have very low noise (≤ 0.04) in both the ON and OFF medication tests, six patients have intermediate values [0.04–0.15], and two patients have very high noise (> 0.16) in both tests. The only exceptions are patients 1 and 8, who exhibit larger changes between the two phases. The large differences among individuals reveal the presence of some subjects with very low exploration tendencies and others with very high ones. This parameter will be linked with the behavioral index “exploration.”
(iv) The upper and lower saturation values for the synapses help the fitting procedure, improving the final cost function, but exhibit rather small ranges (0.7–0.9 for the upper saturation, 0.3–0.45 for the lower one) with no significant differences among groups. In most cases, the values are proximal in the ON and OFF medication cases. There are some exceptions. In particular, some subjects exhibit either a low (0.6) or a high (1.17) upper saturation, indicating a reduced or increased range of synaptic plasticity.
(v) In most cases, the standard deviations of the estimates are rather small, supporting that the procedure can reasonably assess a parameter value with sufficient accuracy.
(vi) The final cost function, reflecting the differences in the correct response cumulative function between the subject and the model, can range from a very low value (in a few cases as low as zero, signifying a perfect superimposition) to a higher but still acceptable value (250–300 revealing a difference of 4–5 responses in the last portion of the cumulative function). The cases with a higher cost function are usually correlated with a high-noise and/or a high learning factor. Indeed, both conditions make it more difficult to make a correct prediction trial by trial.

Finally, to further validate the model's simulation results, we performed the same statistical analysis shown in Fig. 2 (left panels) using the final model scores after training. The results of this analysis, as shown in Figure 2 (right panels), are consistent with those obtained from patients and control subjects. Indeed, a significant difference is observed between the acquisition and reversal phases (Panel C, yellow and orange bars) in the model simulation for PD ON and PD OFF patients. In contrast, no such difference was found in the simulation for controls. Regarding the analysis of the acquisition and reversal phases, no significant difference was found among PD ON, PD OFF, and CS groups.

*Power analysis on the statistical tests* – In order to better contextualize our previous findings, we performed a power analysis by computing the required sample size as a function of the effect size (Cohen’s d), assuming α = 0.05 and a power 1 – β = 0.8, both in the case of a paired and two-group Wilcoxon signed-rank tests [45, 46]. The paired test was applied whenever comparing PD on and PD off, whereas the two-group test was used to compare PDs with CSs. The results, summarized in Figs. S3 and S4 of the supplementary materials, show that a sample size as small as 18 can be sufficient for the paired test if the effect size is greater than 0.55. Conversely, the two-group test requires a much greater size (40 or 50 subjects per group). This result shows that our study may be underpowered to detect subtle but meaningful differences between PD and CS.

Subsequently, we repeated the analysis by computing the effect size (ES) a posteriori from the available data for each test (see Supplementary Materials, Table S3). Only when the effect size was larger than 0.4 did we compute the corresponding a posteriori estimated sample size (with power 1 – β = 0.8 and α = 0.05).

In all tests that provide a statistically significant result, the sample size is adequate (marked in green). In other cases (marked in yellow), subtle statistically significant differences can remain undetected due to an insufficient sample size (in particular, differences between CS and PD ON during reversal, differences in the noise level, and, hence, exploration between CS and PD subjects, especially between CS and PD ON). These differences will be analyzed in a further paper with a wider sample.

### Examples of Model fitting on Individual Curves

In this section, we present and discuss three individual cases characterized by different parameter combinations. They are representative of the differences among individual responses and highlight the relationship between subject behavior and parameter values. In each figure, the participant's behavior (cumulative function of the correct responses) is represented in black, the model’s behavior is in red, and the distance between the two is in blue. Moreover, punishments received by the patient are marked with a black P, while those received by the model are indicated with a red cross. It is worth noting that, throughout the entire test, we provided the same feedback to the model as we did to the patient. Differences in rewards or punishments are a consequence of dissimilar behavior (differences in correct or incorrect choices), as reflected in the cumulative function.

**Fig. 3:**
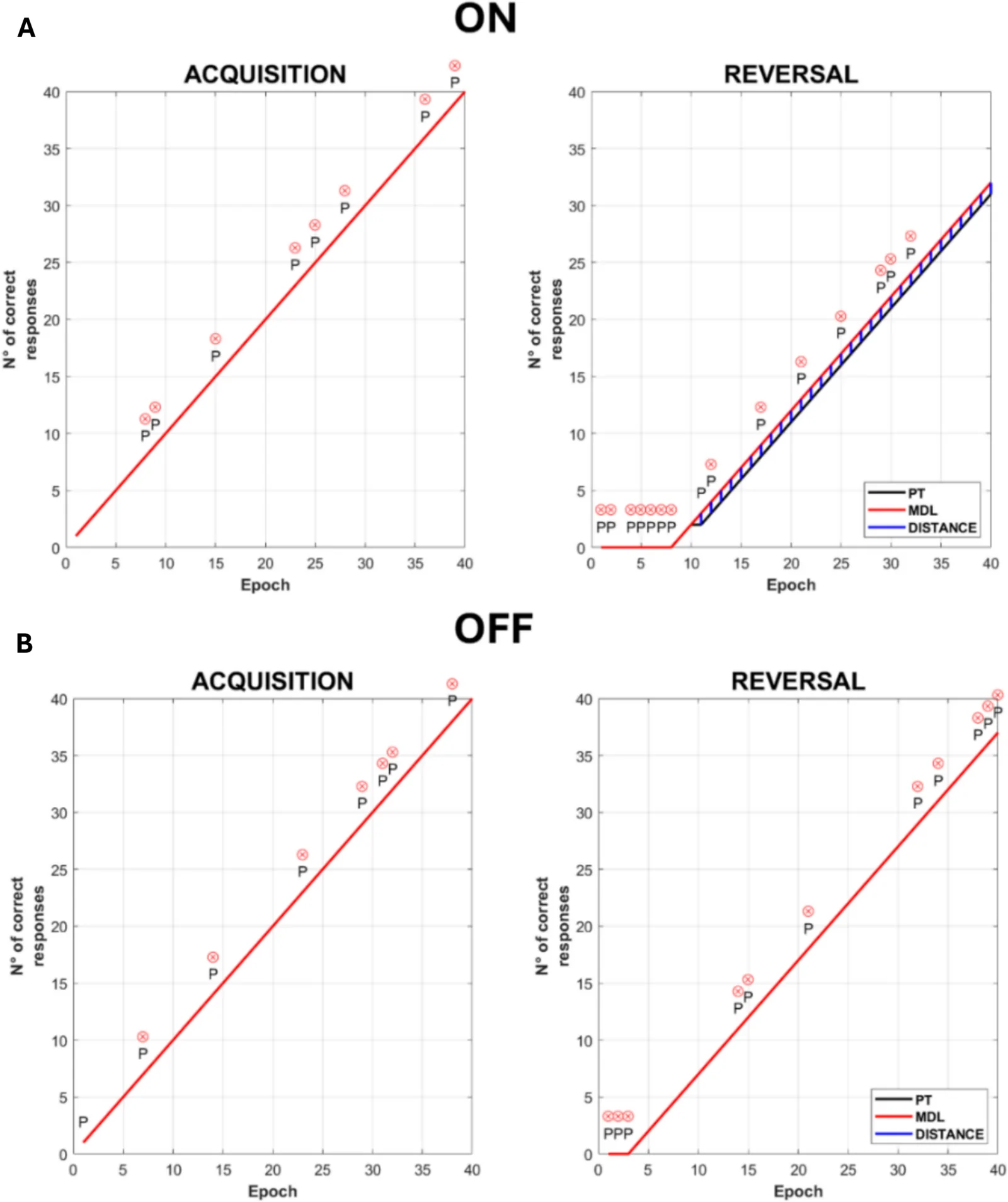
Model representation of Patient 2 with (Panel **A**) and without (Panel **B**) Levodopa medication. The figure illustrates how the model (red line), incorporating dopamine, learning factor, noise, and lower and upper saturations as inputs, replicates the patient’s behavior (black line) in achieving the final score. The model accurately reproduces the patient's performance both during the OFF phase (cost function = 0) and the ON phase (cost function = 30). The parameters for this patient were as follows: in the ON condition, dopamine (1.51), learning factor (1.00), noise (0.02), lower saturation value (0.50), upper saturation value (0.81); and in the OFF condition, dopamine (0.64), learning factor (1.00), noise (0.03), lower saturation value (0.40), upper saturation value (0.78)

Figure 3 illustrates the behavior of patient 2. This patient exhibits low/normal learning factor values (1.0 both ON and OFF medication), small noise (0.02 and 0.03), and rather normal saturation values. As a consequence, his behavior is quite normal. The model predicts this very well, as evidenced by the minimal distance between the two cumulative functions in the model and the participant. The final score during acquisition is 40 in both the ON and OFF medication conditions, signifying no error from the very beginning. As expected, the patient makes some errors at the beginning of the reversal phase. In particular, the patient experiences greater difficulty during the reversal phase in the ON state (dopamine = 1.51), as reflected by a higher number of punishments in the first reversal epochs. As a consequence, the final scores after reversal are 31 and 37 in the ON and OFF medication periods, respectively.

In conclusion, the patient exhibits minimal noise, indicating a strong reliance on previously learned information rather than exploratory behavior. Moreover, the learning factor remains within the normal range, or is rather low, meaning that multiple consecutive punishments must occur before the patient adjusts his/her decision-making. As a result, the learning curve appears linear and rigid, reflecting a deterministic approach to adaptation rather than a flexible response to new feedback.

**Fig. 4:**
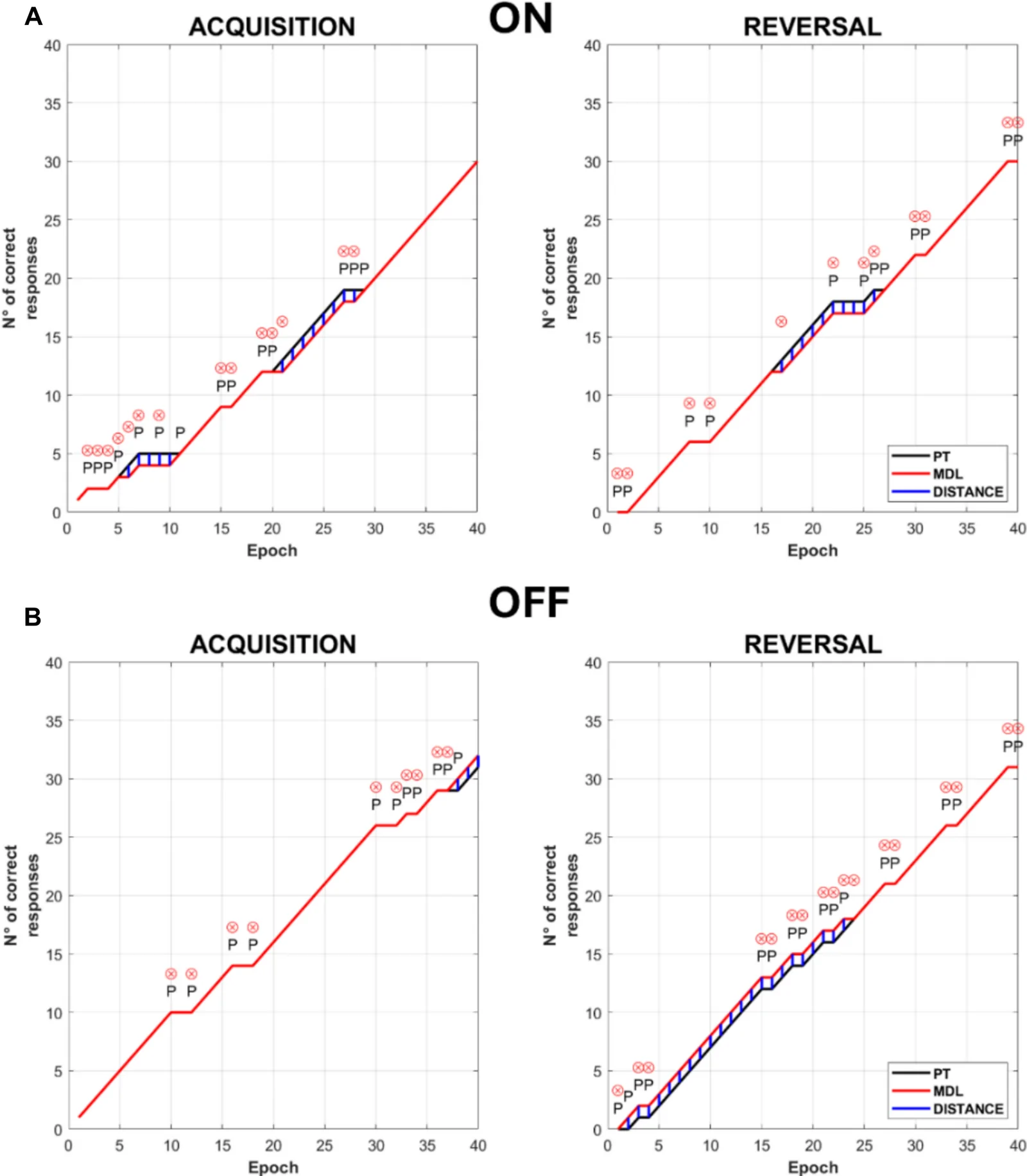
Model representation of Patient 14 with (Panel **A**) and without (Panel **B**) Levodopa medication. The red line represents the model, which closely follows the patient's performance, achieving the same final scores in both ON (acquisition and reversal final scores = 30) and OFF conditions (acquisition and reversal final scores = 31). Model parameters: ON: dopamine (1.20), learning factor (2.30), noise (0.04), lower saturation value (0.50), upper saturation value (0.72). OFF: dopamine (0.65), learning factor (3.00), noise (0.03), lower saturation value (0.50), upper saturation value (0.80). Compared to Patient 2 (Fig. 3), a higher learning factor, lower dopamine level, and increased noise result in faster reversal, more exploration, and a greater response shift following punishment

A distinct behavioral pattern is observed in patient 14 (Fig. 4). This patient exhibits a very high learning rate (2.3 and 3 in the ON and OFF medication conditions, respectively) but small noise (0.04 and 0.03). As a consequence, he/she is extremely prone to punishments. In this case, too, the model can fit the cumulative curve very well. As shown in Figure 4, the patient almost always changes his/her response after a random punishment, which frequently leads him/her to adjust his/her decisions, resulting in a highly reactive, fluctuating learning pattern. As a consequence, the final scores are worse than in the previous case. The patient shows no clear distinction between the acquisition and reversal phases, with only minimal differences between the ON and OFF medication conditions. However, performance is slightly better in the OFF state (final scores 30 − 30 in the ON state, 31 − 31 in the OFF state).

An example of a patient with both very high noise and high learning factor (Patient 3) is shown in the Supplementary Materials (Fig. S5).

**Fig. 5:**
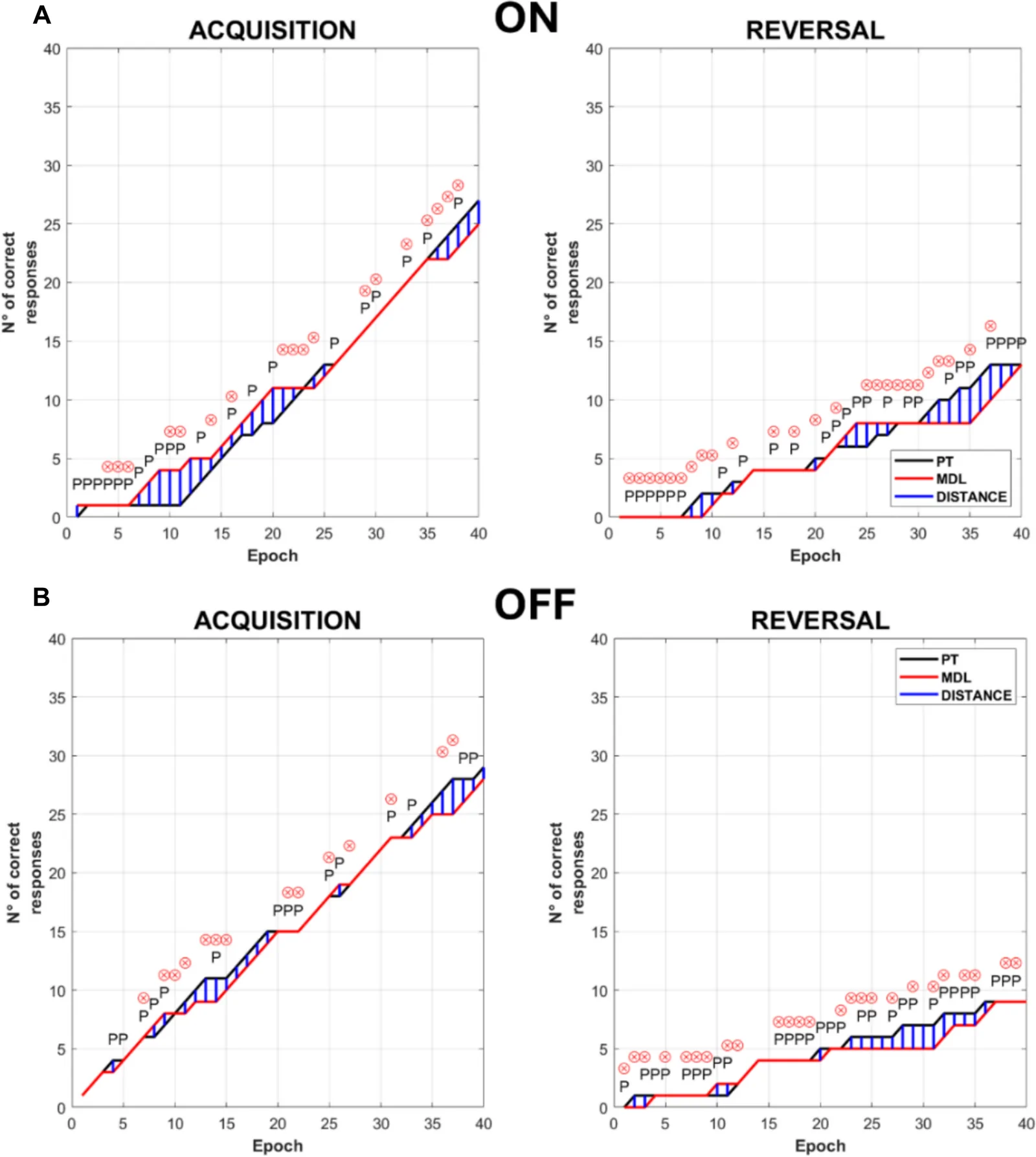
Model representation of patient 18 with (Panel **A**) and without (Panel **B**) Levodopa medication. Model parameters: ON: dopamine (2.60), learning factor (1.60), noise (0.27), lower saturation value (0.37), upper saturation value (0.77). OFF: dopamine (0.60), learning factor (1), noise (0.27), lower saturation value (0.40), upper saturation value (1.17). Patient 18 is characterized by a lack of proper response to punishment, remaining focused on the initial decision without modifying it. The reversal phases in both ON and OFF medication conditions involve many errors, resulting in very low final scores: 13 and 9, respectively, less than 50% of the maximum possible score. The model representation incorporates high dopamine levels and noise during the ON phase, resulting in excessive exploration and a strong commitment to the initial decision. In the OFF phase, the model receives high-noise input and an elevated saturation value, reducing its ability to learn from punishments during the reversal phase

The last patient examined here is patient 18 (Fig. 5), who exhibits different behavior and parameter estimates compared with the previous ones. In fact, the learning rate is rather normal, with values of 1.6 and 1.0 in the ON and OFF medication states, respectively; however, the noise level is high, at 0.27 in both states. This high noise leads to the exploration of new answers, even without receiving a punishment, which explains the low-to-average final scores during the acquisition phase (29 and 27 in the OFF and ON medication cases, respectively). Moreover, this patient exhibits distinct compromised reversal phases in OFF and ON states. He/she exhibits a high upper saturation (1.17) in the OFF medication, which is reflected in difficulty changing opinion during reversal. Due to the high level of synapses reached in the previous acquisition phase, this patient persists in his/her initial choice without willingness to change it (with final scores as low as 9 in OFF medication), a behavior that the model can simulate rather well with the estimated parameter values. Differently, in the ON medication case, the patient exhibits a very high estimated DA level (2.60), which produces insensitivity to punishments and a bad reversal score (13) according to an overdose hypothesis.

**Fig. 6:**
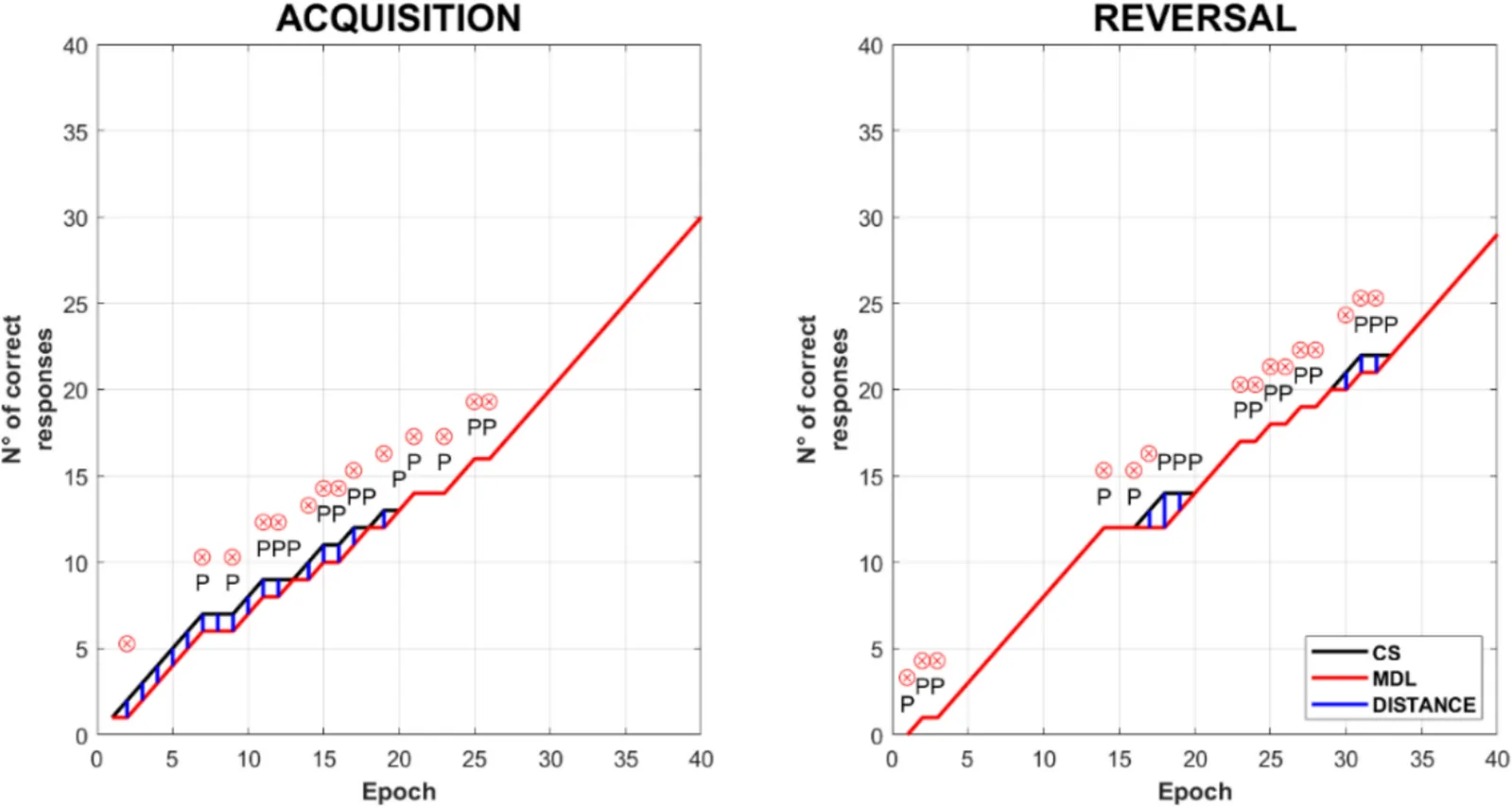
Model representation of Control 2. Model parameters: dopamine (1), learning factor (3.86), noise (0.04), lower saturation value (0.38), upper saturation value (0.68). The model follows the choice trajectory of the control subject quite well, ascribing them to a very high learning factor; the subject often modifies the response after punishment. Additionally, the low noise level prevents exploratory behavior

Finally, Fig. 6 gives one example of a control subject. The participant has a dopamine level of approximately 1 (Table S2). The subject was simulated with a very high learning factor (3.86), which enabled the model to accurately reproduce the frequent decision changes following a punishment. A noise level of 0.04 results in relatively limited exploratory behavior by the model.

### Correlation Between the Parameters and the Final Score

**Fig. 7:**
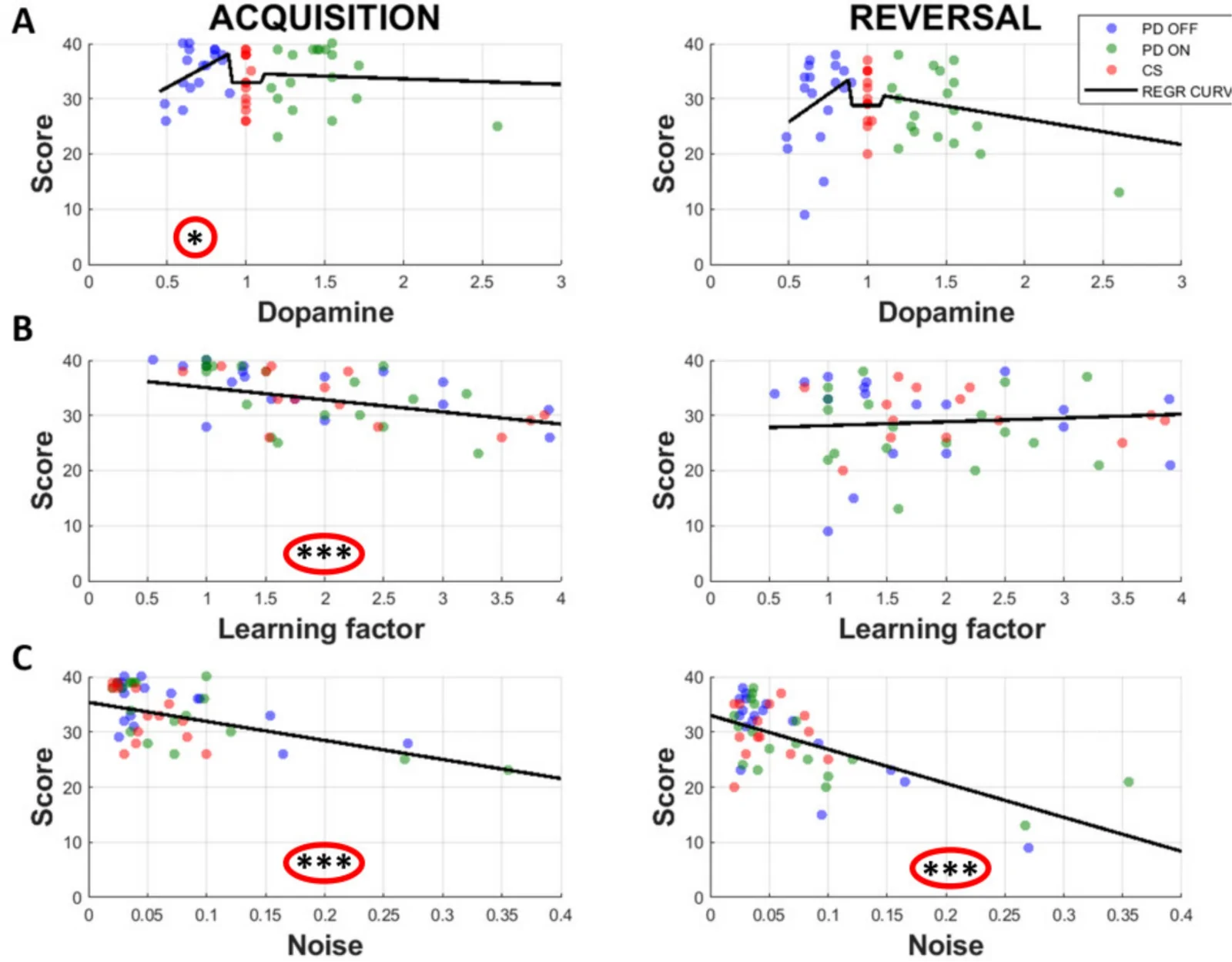
Relationship between model parameters and final scores in the acquisition phase (first column) and the reversal phase (second column). Panel **A** highlights a positive relationship between the dopamine levels and final scores, both in acquisition and reversal, for patients without medication (PD OFF). In contrast, a negative relationship is observed for those under Levodopa medication (PD ON), especially in the reversal condition. Panel **B** shows that a high learning factor can negatively impact performance in the acquisition phase. Panel **C** depicts the strong interdependence between high-noise values and the learning curve. Statistical results for regression coefficients are shown in the form of asterisks (*: 0.01 ≤ *p* ≤ 0.05,**: 0.001 ≤ *p* ≤ 0.01, ***: *p* ≤ 0.001), as determined using a two-tailed t-test

Figure 7 displays the results of the multiple correlation analysis between the estimated parameters and the final score. This analysis is presented separately for the acquisition and reversal phases. Moreover, only for tonic dopamine, the only parameter showing significant differences among the three cases, the analysis is performed separately for PD ON patients (green), PD OFF patients (blue), and CS (red), with the regression lines plotted in black. For the other important parameters (learning rate and noise), we computed a single regression for the three cases.

The dopamine regression curve (Panel A) shows a positive relationship in PD OFF patients across both the acquisition and reversal phases, indicating that scores improve as dopamine levels increase (this effect is statistically significant in the acquisition phase). For CS, the values remain constant, as they are simulated in the model with a dopamine level of approximately 1. In contrast, PD ON patients exhibit a slightly negative relationship, especially during reversal, meaning that as dopamine increases, their scores decrease. This conforms with the literature's findings (see Discussion). This regression curve is not very steep, as only a few participants have a very high dopamine estimate in the ON phase. A stronger relationship can be observed if single regression (instead of multiple regression) is performed (see Supplementary Materials Figure S6).

Regarding the learning factor (Panel B), in the acquisition phase, a lower value enables patients to achieve a higher overall score (a negative statistically significant relationship). In contrast, during the reversal phase, a positive but non-significant relationship is observed.

Finally, considering the noise (Panel C), high values greatly disadvantage both the acquisition and reversal phases for all subjects. The steep curve is highly statistically significant.

Figure S6 of the Supplementary Materials shows the results of the single regression, performed on parameters separately. These findings further emphasize the negative effect of high dopamine levels during the reversal phase.

### Relationship Between Behavioral Indices and Model Parameters

In the method section, we described two behavioral indices derived from participant responses: punishment sensitivity, which summarizes how much a participant learns from mistakes, and exploration, which reflects the tendency to change a choice even in the absence of an immediate punishment. Here, we test the assumption that the first index is correlated with the model learning factor, and the second index is correlated with noise. To this end, we computed regression curves linking the estimated parameter to the behavioral index for each participant.

**Fig. 8:**
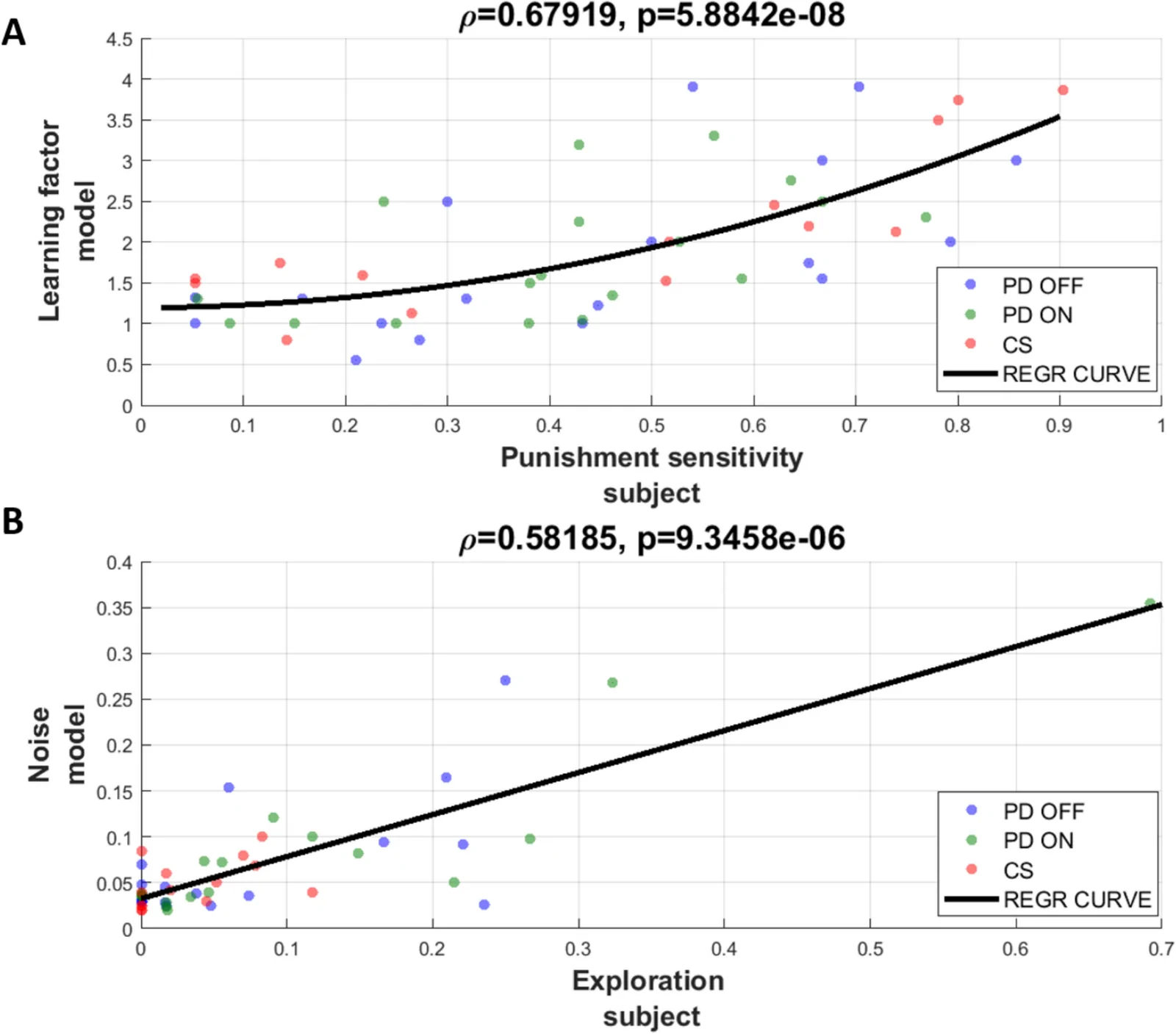
Relationship between model parameters and subject behavioral indices. Panel **A** represents the relationship between the learning factor and the punishment sensitivity (computed as the number of changes in subject response after a punishment over the total number of punishments received). Panel **B** illustrates the relationship between the noise SD of the model and the exploration performed by the subject (computed as the number of changes in subject response after rewards divided by the total number of trials rewarded). The strength and direction of the association are quantified using Pearson’s correlation coefficient (*ρ*), displayed in the figure’s title along with the corresponding *p*-value. A regression curve is presented in both panels: parabolic for punishment sensitivity and linear for noise, visually representing the trends in the data

Figure 8a shows a strong nonlinear correlation between punishment sensitivity and the learning factor, characterized by a convex parabolic relationship with Pearson's correlation coefficient *ρ* = 0.68.

Noise exhibits a good linear correlation with the subject exploration, with a Pearson's correlation coefficient of *ρ* = 0.58 (Fig. 8b).

These behavioral indices can provide a basis for a deeper understanding of the participant's capacity for action choice, as they can establish how much a subject is influenced by the last punishment received and how much is inclined to explore new choices.

## Discussion

The present work demonstrates the feasibility of using a neurocomputational model of the BG with Hebbian learning to simulate individual behavior during a two-choice probabilistic task comprising both direct and reversal phases in patients with Parkinson's disease and control participants. Learning in the model is governed by phasic dopamine responses to unexpected rewards and punishments.

A first important achievement is that the model can reproduce the different patterns observed experimentally (both in PD subjects and control participants) ascribing the observed differences to a combination of three main mechanisms: i) the dopamine tonic level, which sets a different baseline for the Go and NoGo pathways and a different sensitivity to rewards and punishments; ii) the values of plastic synapses in the striatum, consequent of previous learning. These values determine how much a participant can leverage prior knowledge. To describe this mechanism, three parameters have been estimated, representing the lower and upper saturation values for the synapses and the Hebbian learning factor. High and low values of saturation indicate that the participant is strongly influenced by previous experience (hence exhibits a poor reversal). The learning factor is particularly important, as it describes how many experiences are required to achieve correct behavior and how much recent outcomes can influence a participant. In particular, high learning factors imply that a participant can rapidly modify their previous choice even after a single punishment, resulting in a poor cumulative function; and iii) the tendency of a participant to explore new possibilities, which is described in our model with a zero-mean white noise superimposed on the cortical activity. High values of SD for the noise induce continuous exploration, overcoming the exploitation of past experience and thus reducing the cumulative function. In the case of extremely high noise, the response becomes almost random, resulting in approximately 50% correct responses.

We demonstrated that, with an appropriate combination of these mechanisms (resulting in five parameter estimates), all different individual patterns could be reproduced quite well. Moreover, in most cases, the parameter estimates exhibit small standard deviations and are similar within the same PD patient during the ON and OFF medication sessions, confirming the reliability of the present approach. The only clear difference between the ON and OFF medication phases is tonic DA levels, which are consistently higher in the ON phase and lower in the OFF phase.

More specifically, from the present trials, various distinct behavioral responses emerge, characterized by distinct patterns of the cumulative function (i.e., the progressive sum of correct responses). Some participants (as the one displayed in Fig. 3) exhibit almost no error during the direct learning phase (considering that the first choice is always initially rewarded and assumed to be correct), but require a few epochs to invert the choice at the beginning of the reversal phase. This is the typical behavior when a moderate learning rate (i.e., recent results do not excessively influence the subject) and a moderate level of exploration are present. However, these subjects require more time to find a correct reversal phase and are not very flexible.

A second typology, as illustrated in Fig. 4, exhibits a much higher learning capacity and is therefore more influenced by recent random events (e.g., the 20% of punishments after a correct choice); i.e., subjects frequently change their strategy even after a single punishment. As a consequence, the final score is modest, approximately 30 out of 40. However, these participants generally perform better during the initial reversal phase, with higher flexibility.

Another typical subject (see Fig. 5) can be characterized by a normal learning factor but very high synaptic saturation values. This signifies that synapses can reach a very high level at the end of the direct phase. As a consequence, the participant continues to exploit past knowledge with almost no flexibility in the reversal phase: He/she remains stuck on the previous choice, with a score during reversal much smaller than 50% (approximately 9/40). Alternatively, a very high level of DA (as in patient 18 in Fig. 5 during the ON condition) can also explain a poor reversal, since high DA levels produce reduced sensitivity to punishment.

Finally, we found a few subjects with very high noise levels: Their behavior is strongly explorative, with almost no exploitation of previous learning, and they exhibit quite a random pattern of responses with a final score (both in the direct and reversal phases) close to 50% (20/40) (an example of the latter typology is patient 3 of Table S1, shown in Supplementary materials).

Of course, these typologies are just paradigmatic. Since parameters can be combined in different ways, alternatives can be mixed, resulting in a wider spectrum of behaviors.

It is worth noting that our final cost function is always satisfactory but, of course, exhibits a larger value (indicating greater differences between the model and participant responses) in the presence of large noise, i.e., during clear exploratory behavior. In fact, the individual responses during exploration are, by their nature, unpredictable a priori; in this condition, the model can exhibit a similar random exploration (that is, a change in the preferred choice even after a reward), but this can occur at different moments compared with those of the participant. What is important is the final score, which reflects the overall behavior – that is, how much exploration the participant conducted during the forty trials.

An important support to the previous ideas is provided by the regression analysis, which substantiates the role of the main parameters, and, above all, by the strict relationship we found between some parameters and behavioral indices. It is worth noting that, for simplicity, we focused on the three most important parameters (tonic DA, learning factor, and noise SD) during these analyses, without considering synapse saturation to make the results clearer.

The regression analysis confirms that a very high learning factor reduces performance during the direct phase, increasing sensitivity to punishments, but may play a mild positive role during reversal (although not statistically significant), favoring a rapid change in preferred contingencies. Large noise has a detrimental effect on both the direct and reversal phases, indicating excessive pathological flexibility. The role of tonic DA is clear in the OFF condition: Patients with very low DA perform badly, since they exhibit the typical poor response of severe Parkinsonism. Patients with higher DA perform better during the OFF condition, resulting in a positive correlation between DA levels and performance. However, on average, OFF PD patients perform similarly to control subjects, as several control individuals also exhibit poor performance (see Fig. 7, upper left panel).

The role of tonic DA in ON conditions (i.e., after Levodopa medication in PD patients) warrants a deeper discussion. Statistically, no differences are evident between OFF, ON, and control participants in our work during either the direct or reverse phases. The absence of differences in the direct phase is consistent with the literature and is evident in Figs. 2, 4, and 7. Conversely, the absence of statistical differences during the reversal phase is at odds with some data in the literature [9, 12, 47]. In particular, [9] and [47] suggest that ON patients exhibit a worse reversal than OFF patients due to lower sensitivity to punishment. These differences in reversal were also evident in our previous simulations [21], performed using high DA levels to simulate the ON condition. Indeed, looking at our results, we can observe that PD ON patients have on average a worse score in the reversal phase (Fig. 2a and b) and that high tonic DA levels reduce the reversal performance, especially when the estimated DA level is very high, resulting in a negative correlation (Figure 7, upper right panel; see also the patient in Figure 5, who exhibits very high DA in the ON medication state). A negative correlation between DA level and reversal score becomes even more evident when a single regression is performed rather than a multiple regression (see Supplementary Materials, Fig. S6). However, neither of these results reaches statistical significance in the present work.

There are two main reasons for the absence of significant reversal learning deficits in the PD ON: i) Most patients did not receive a strong levodopa dose in this study. Indeed, we aimed to establish whether patients can be clinically characterized even at an early stage of the pathology. Accordingly, only three patients have an estimated value higher than 1.7. Two of them exhibit a poorer response in the reversal phase during ON medication compared to the OFF condition, and the other exhibits a dramatically poorer reversal in both ON and OFF medication conditions. Hence, we can conjecture that the absence of a clear negative effect of high DA levels during reversal may be due to the patients’ cohort used in this study, who only rarely adhere to the overdose [12]. This may be the reason why the effect size between PD ON and PD OFF in the reversal learning was so small (about 0.2, See Supplementary Materials); ii) the limited number of patients, which reduces the effective power of our test (see also the analysis in section Results), especially when the effect size is so small.

Regarding the estimated tonic DA, our values are consistently higher in the ON condition than in the OFF condition. At the same time, control participants can always be simulated using an intermediate tonic DA level close to the basal one. These results support the reliability of the estimated DA levels.

Regarding the learning rate and the noise SD, we found a significant relationship between the estimated parameters and two original behavioral indices: punishment sensitivity and exploration rate. To our knowledge, this is the first time these indices have been proposed and related to a mechanistic neurocomputational explanation.

The learning factor is correlated with the sensitivity to punishments; this relationship is nonlinear and becomes steeper at high sensitivity values. The exploration index exhibits a pretty good linear correlation with the estimated SD of noise. The values of the correlation coefficient in Fig. 8 appear moderate (r = 0.68 and r = 0.58, respectively), but this was well expected; in fact, the total behavior results from the combination of several mechanisms acting together, summarized with five parameters; hence, other factors can interact with the observed relationship, making it rather noisy. Interestingly, the behavioral indices (punishment sensitivity and exploration) and the related parameter values (learning rate and noise SD) do not result significantly different among the three groups. We can conclude that Parkinsonism does not directly affect these mechanisms; its effects primarily result from altered DA levels. However, these findings can be affected by the limited statistical power of our tests, as discussed in the Supplementary Materials.

The previous results support the idea that our estimated parameters are not merely the consequence of a mathematical black-box procedure designed to fit curves, as is often the case with most AI algorithms. Still, they are related to actual clinical conditions (the estimated DA) and the participants' specific behavioral characteristics. This result opens up a possible perspective for the future use of the model and the related fitting procedure in a clinical setting, characterizing each individual patient with a few parameters that have clear meaning. Furthermore, the present work emphasizes the value of neurocomputational BG models for elucidating the various factors involved in adaptive behavior and flexibility, a topic with significant psychological and neurological implications.

It is important to emphasize that the present work does not aim to explore new mechanisms, but rather to assess whether individual differences among subjects can be captured using a previous model and by estimating only a small number of parameters with a clear relationship to behavior. To our knowledge, this represents a novel contribution. We are not aware of any previous models that reproduce individual variability in PD by relating it to a limited number of neurologically grounded factors. We claim this is a fundamental advancement over previous mechanistic insights, which have been almost exclusively devoted to population behavior and differences between pathological and non-pathological groups.

Furthermore, this individual characterization can be valuable in future clinical practice for designing a customized rehabilitation protocol for PD patients.

More specifically, our individual indices of punishment sensitivity and exploratory attitude consistently reflect how patients respond to feedback across different learning contexts, independent of their dopaminergic state, and hence may be exploited to optimize a rehabilitation procedure. In this context, patients with high sensitivity to punishment (hence, a high learning factor) may benefit from rehabilitation approaches characterized by reduced ambiguity and attenuated negative feedback, thereby limiting excessive strategy switching. On the other hand, patients with a predominance of an exploratory attitude (hence, high noise) may require more salient cues and greater contextual variability to promote behavioral updating.

Although these considerations require further validation, the present results suggest that computational parameters derived from reinforcement learning with reversal tasks may provide informative markers for cognitive stratification beyond the conventional ON–OFF framework and may inform the development of more tailored rehabilitative approaches.

Future models' use can also contribute to understanding different pathologies (such as Tourette syndrome or Attention Deficit Hyperactivity Disorder) characterized by altered DA levels in the BG and by behavioral imbalances.

### Limitations and Future Directions

i) The Hebbian rule used in this work involves a synaptic potentiation when both the pre-synaptic and post-synaptic neurons are inhibited. This assumption appears to be at odds with physiological experiments on excitatory synapses. However, as described in the Method section and in Figure S2 of the Supplementary Materials, and as already discussed in our previous work [21], this can be justified by the presence of active inhibitory interneurons in the striatum.

Many experimental results support the presence of inhibitory interneurons in the striatum [48] and their involvement in learning. Recently, Lee et al. [49] demonstrated that a disynaptic inhibitory circuit couples parvalbumin interneuron cells to projection neurons in the striatum and that these interneurons selectively enhance performance early in learning. Data from Boroujeni et al. [50] suggest that fast-spiking interneurons in the striatum, which receive prominent cortical inputs and impose feedforward inhibition on large ensembles of spiny projection neurons, exert a strong influence during learning. In a recent study, Rotariu et al. [51] proposes that local inhibitory interneurons shape early representations and influence task performance in the dorsal medial striatum. The authors identify the high plasticity of feedforward inhibition as a critical modulator of striatal projection firing activity and early learning.

A possible improved version of our model might include inhibitory interneurons directly and implement an updated version of the Hebb rule.

ii) A limitation of this work is that we did not test our results (i.e., the reliability of parameter estimates) on a second independent dataset, as is usually done in machine learning algorithms. Of course, since our results focus on individual parameter estimates, a second independent dataset should be obtained from the same subjects and, possibly, on the same days, which was not possible under the present clinical protocol.

A more complete validation can be achieved in future work by performing several different tasks on the same subject, estimating parameters only on some tasks, and using the remaining ones to test the model’s capacity. A possibility may be to perform several probabilistic reversal tasks with different reward probabilities (for instance 70%, 80%, 90% and 100%), or to alternate a probabilistic reversal task with other tasks designed to test learning and flexibility, such as a task-switching protocol [52], or designed to evaluate motor behavior (such as the alternate finger tapping task, already tested in previous works of us [53]).

iii) It is worth noting that, as shown in Supplementary Materials, our statistical analysis may be underpowered to detect subtle differences between control and PD subjects. While detecting these differences was not the aim of the present work, they can be the target of a future, more clinical work based on a larger dataset (in preparation).

## Supplementary Information

Below is the link to the electronic supplementary material.
Supplementary file (DOCX 235 kb)
