# Supplementary material for: A Mechanistic Understanding of the Different Factors Affecting Adaptation and Flexibility During Probabilistic Reversal Learning: A Neurocomputational Study on Patients with Parkinson’s Disease and Control Subjects
Source: Ann Biomed Eng. 2026 Mar 4;54(9):3192–212. doi: 10.1007/s10439-026-04038-4 (PMC13522018; doi:10.1007/s10439-026-04038-4)
Supplement: Supplementary file 1 [file 10439_2026_4038_MOESM1_ESM.pdf]

SUPPLEMENTARY MATERIAL

A mechanistic understanding of the different factors affecting adaptation and flexibility during probabilistic reversal learning: a neurocomputational study on patients with Parkinson's Disease and control subjects.

Giulia Piermaria[1], Silvana Pelle[1], Alessio Zizzi[2], Carola Cosentino[2], Susanna Mezzarobba[2,3], Giovanna Calandra-Buonaura [4,5], Luisa Sambati[5], Federica Scarpellini[4], Elisa Pelosin[2,3], Mauro Ursino[1*]

1 - Department of Electrical, Electronic, and Information Engineering "Guglielmo Marconi", University of Bologna, 47521 Cesena, Italy

[2] Department of Neuroscience, Rehabilitation, Ophthalmology, Genetics and Maternal Child Health, University of Genoa, 16132 Genoa, Italy
[3]IRCCS Ospedale Policlinico San Martino, Genoa, Italy

[4]Department of Biomedical and NeuroMotor Sciences, Alma Mater Studiorum –University of Bologna, Italy
[5]IRCCS, Istituto delle Scienze Neurologiche di Bologna, Italy

*** Corresponding Author:** Mauro Ursino - Department of Electrical, Electronic and Information Engineering "Guglielmo Marconi" - University of Bologna - Area di Campus Cesena, Via Dell'Università 50, I 47521 Cesena, FC

Email: mauro.ursino@unibo.it

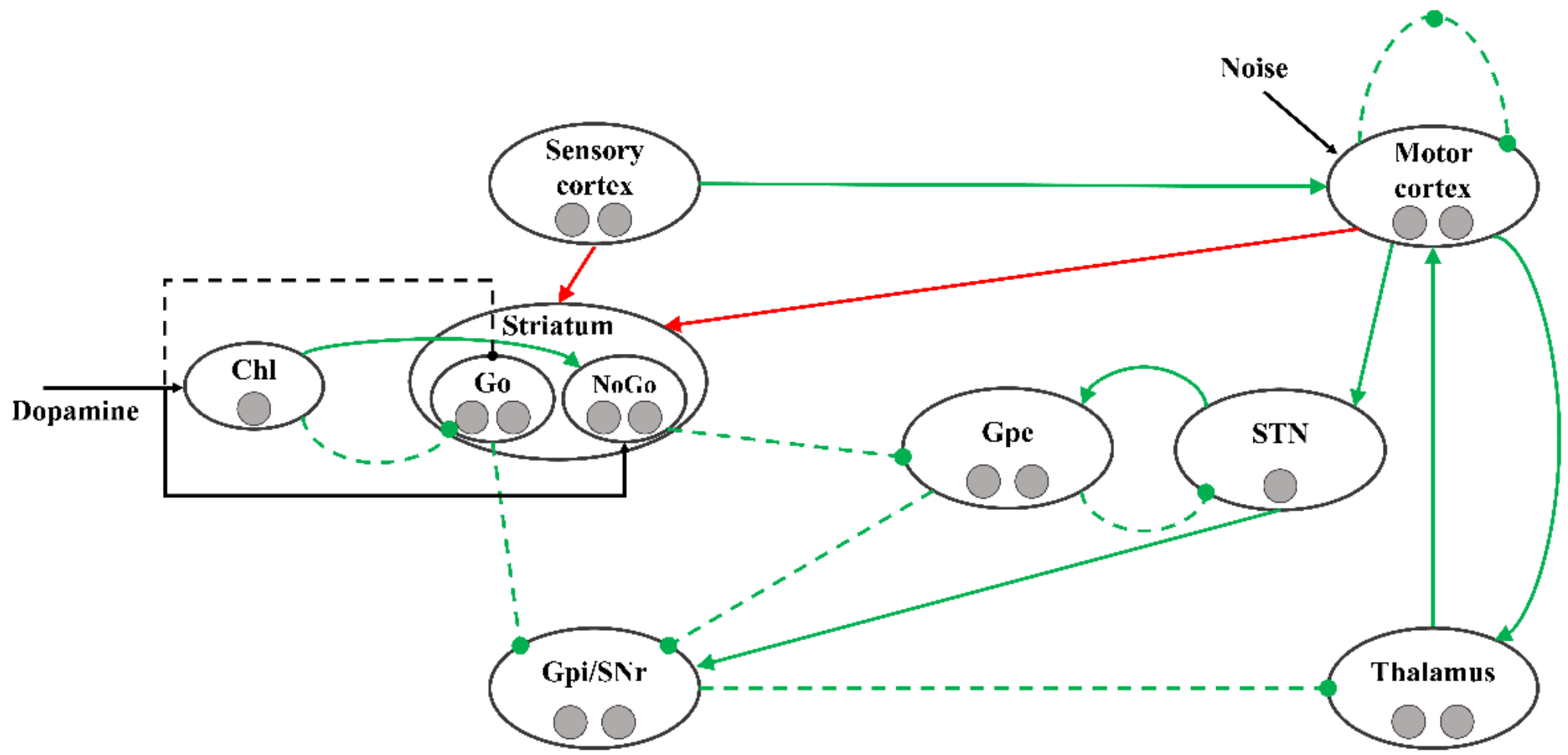

Fig. S1 - Block diagram describing the principal regions of the BG model and their relationships. Ovals represent different areas, while gray circles represent neurons. Continuous arrow-shaped arrows represent excitatory synapses, and dashed, circle-shaped arrows represent inhibitory synapses. Red arrows are trainable synapses, green arrows represent fixed synapses, and black arrows represent external inputs. The model includes the globus pallidus pars externa (Gpe), the globus pallidus pars interna (Gpi), the cholinergic area (Chl), subthalamic nucleus (STN), and the Substantia Nigra (SNR).

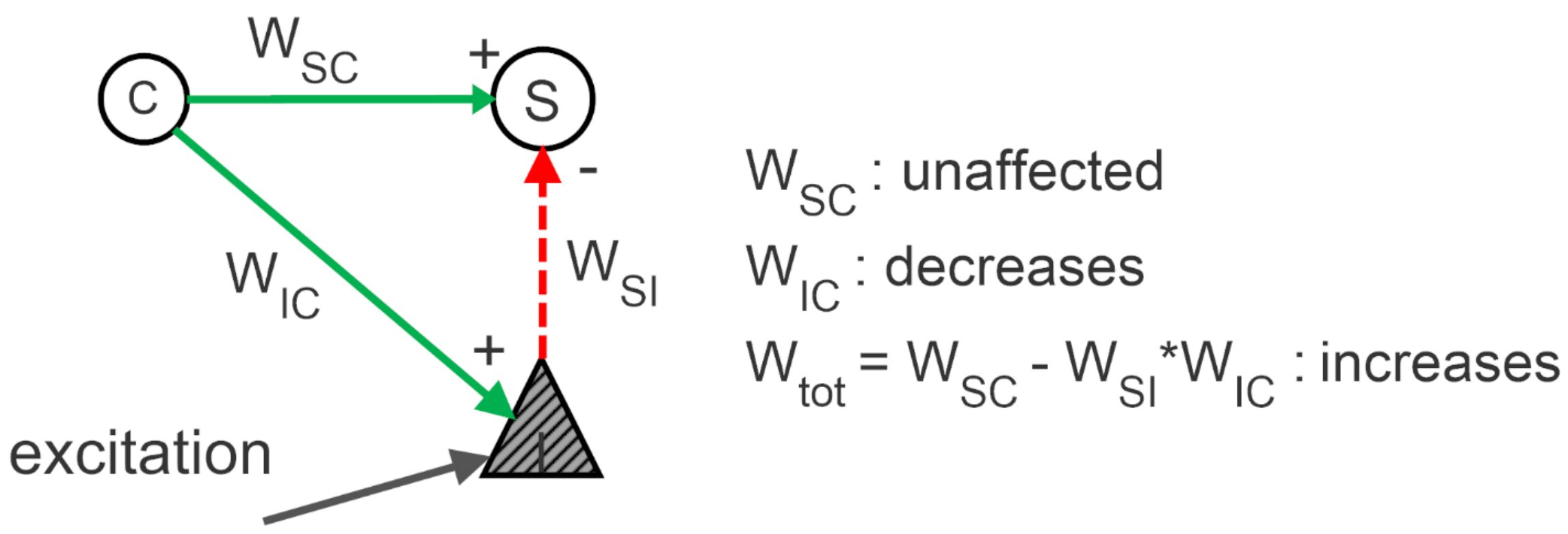

Fig. S2 – An example of how a Hebbian synapse can be increased when both the pre-synaptic and post-synaptic neurons are below the threshold, thanks to the presence of an intermediate inhibitory interneuron. Green lines represent excitatory synapses; red lines are GABAergic inhibitory synapses. The meaning of the symbols is – C: cortical neuron; S: striatal output neuron; I: striatal interneuron. The total synapse (named Wtot) results from a balance between an excitatory synapse (named WSC) and a bi-synaptic inhibitory link (WSI in series with WIC). Assuming approximately linear behavior, we have: Wtot = WSC – WSI*WIC. Moreover, let us assume that the two excitatory synapses are plastic according to the Hebbian rule, including long-term potentiation and heterosynaptic and homosynaptic long-term depression.

Let us assume a condition in which both the cortical neuron (presynaptic) and the striatal projecting neuron (postsynaptic) are inhibited (open circles represent neuron activity below the threshold), whereas the inhibitory interneuron is moderately active (dashed fills indicate that the neuron is excited above the threshold). In this condition, synapse WIC experiments heterosynaptic depotentiation, resulting in a global increase in the total synapse Wtot.

	DOPAMINE		LEARNING FACTOR		NOISE		LOW SAT		UP SAT		COST FUNCTION		FINAL SCORE				FINAL MDL SCORE			
	ON	OFF	ON	OFF	ON	OFF	ON	OFF	ON	OFF	ON	OFF	ON		OFF		ON		OFF	
													ACQ	REV	ACQ	REV	ACQ	REV	ACQ	REV
	***												**		**		**		**	
**PT1**	1.72 (3.90E-02)	0.60 (4.77E-02)	2.25 (1.31E-01)	1.75 (2.30E-01)	0.10 (8.39E-03)	0.04 (3.94E-03)	0.45 (1.82E-02)	0.45 (7.77E-03)	0.70 (2.33E-02)	0.60 (9.84E-03)	302	85	32	19	34	32	36	20	33	32
**PT2**	1.51 (2.40E-02)	0.64 (0.00E+00)	1.00 (1.83E-01)	1.00 (0.00E+00)	0.02 (1.94E-02)	0.03 (0.00E+00)	0.50 (1.23E-01)	0.40 (0.00E+00)	0.81 (1.27E-02)	0.78 (0.00E+00)	41	0	40	31	40	37	39	31	40	37
**PT3**	1.20 (3.35E-01)	0.49 (3.36E-02)	3.30 (8.94E-01)	3.91 (1.20E-01)	0.36 (8.06E-02)	0.16 (1.30E-02)	0.35 (7.65E-02)	0.42 (2.75E-02)	0.80 (6.06E-02)	0.83 (1.13E-02)	341	95	20	19	26	19	23	21	26	21
**PT4**	1.70 (1.87E-02)	0.85 (3.03E-02)	2.00 (1.32E-01)	1.31 (6.88E-02)	0.12 (7.55E-03)	0.05 (4.07E-03)	0.43 (1.33E-02)	0.40 (1.75E-02)	0.70 (9.05E-03)	0.80 (2.55E-02)	65	65	30	24	40	35	30	25	38	35
**PT5**	1.20 (7.79E-02)	0.64 (8.21E-03)	1.30 (6.12E-02)	1.31 (2.32E-02)	0.04 (1.07E-02)	0.03 (8.99E-04)	0.40 (2.88E-02)	0.45 (4.10E-03)	0.83 (4.26E-03)	0.80 (4.10E-03)	39	8	40	38	39	33	39	38	39	34
**PT6**	1.30 (1.12E-01)	0.80 (4.49E-03)	1.50 (2.33E-01)	1.00 (6.67E-02)	0.03 (1.10E-02)	0.03 (3.17E-03)	0.40 (6.35E-02)	0.40 (1.87E-02)	0.80 (1.00E-02)	0.78 (4.52E-02)	181	96	38	25	40	31	38	24	39	33
**PT7**	1.30 (4.72E-02)	0.75 (8.41E-02)	2.50 (6.13E-02)	3.00 (2.37E-01)	0.05 (1.50E-04)	0.09 (6.12E-03)	0.45 (2.17E-02)	0.50 (9.04E-03)	0.75 (1.46E-02)	0.86 (1.62E-02)	148	170	27	27	35	24	28	27	36	28
**PT8**	1.55 (2.30E-02)	0.63 (4.53E-02)	1.55 (2.09E-02)	1.33 (1.93E-01)	0.07 (1.72E-03)	0.03 (6.62E-03)	0.40 (5.58E-03)	0.40 (5.75E-02)	0.68 (8.36E-03)	0.75 (1.06E-01)	50	76	26	28	40	37	26	28	37	36
**PT9**	1.55 (9.06E-02)	0.73 (7.53E-03)	1.00 (1.20E-01)	1.22 (1.85E-01)	0.10 (4.98E-03)	0.09 (1.64E-02)	0.40 (1.98E-02)	0.45 (1.24E-02)	0.75 (3.61E-02)	1.00 (1.43E-02)	133	263	38	21	36	12	40	22	36	15
**PT10**	1.47 (2.53E-02)	0.60 (4.17E-02)	1.00 (3.17E-02)	0.55 (7.15E-02)	0.04 (4.99E-03)	0.05 (7.20E-04)	0.4 (2.80E-02)	0.4 (6.39E-02)	0.81 (3.16E-03)	0.71 (9.10E-02)	138	68	40	34	40	33	39	35	40	34
**PT11**	1.55 (1.50E-02)	0.48 (3.01E-03)	1.00 (9.11E-03)	2.00 (4.06E-02)	0.02 (5.78E-04)	0.03 (7.31E-04)	0.4 (6.24E-03)	0.40 (1.80E-02)	0.78 (1.19E-03)	0.80 (3.29E-02)	41	275	37	33	30	23	38	33	29	23
**PT12**	1.16 (2.70E-01)	0.70 (1.01E-01)	1.34 (6.97E-01)	1.55 (1.23E-01)	0.07 (1.25E-02)	0.15 (1.10E-02)	0.42 (6.16E-02)	0.40 (3.04E-02)	0.80 (2.92E-02)	0.80 (5.94E-02)	182	70	32	32	34	22	32	32	33	23
**PT13**	1.55 (3.48E-03)	0.90 (4.39E-02)	3.20 (6.83E-01)	3.90 (1.30E-01)	0.04 (2.53E-02)	0.04 (2.25E-03)	0.30 (5.29E-02)	0.30 (1.58E-02)	0.80 (4.53E-03)	0.78 (1.30E-02)	54	154	33	38	29	36	34	37	31	33
**PT14**	1.20 (1.98E-02)	0.65 (1.62E-02)	2.30 (1.41E-01)	3.00 (1.20E-01)	0.04 (1.82E-03)	0.03 (1.05E-03)	0.50 (6.12E-03)	0.50 (1.80E-02)	0.72 (3.86E-03)	0.80 (5.17E-03)	23	25	30	30	31	31	30	30	32	31
**PT15**	1.45 (6.22E-02)	0.80 (4.48E-02)	1.05 (4.58E-02)	0.80 (3.57E-02)	0.04 (1.32E-03)	0.03 (3.33E-04)	0.4 (1.61E-02)	0.45 (1.15E-02)	0.80 (1.49E-02)	0.75 (2.36E-02)	195	26	36	21	38	36	39	23	39	36
**PT16**	1.28 (4.62E-02)	0.85 (1.38E-01)	2.75 (1.23E-01)	2.00 (1.86E-01)	0.08 (9.80E-03)	0.07 (3.79E-03)	0.43 (1.24E-02)	0.30 (4.40E-02)	0.80 (1.10E-02)	0.83 (3.99E-02)	171	194	30	23	38	30	33	25	37	32
**PT17**	1.43 (2.43E-01)	0.80 (5.37E-02)	2.50 (3.46E-01)	2.50 (1.81E-01)	0.04 (3.05E-02)	0.03 (1.55E-03)	0.30 (2.37E-02)	0.40 (1.48E-02)	0.80 (1.30E-02)	0.80 (3.94E-03)	30	60	39	36	39	37	39	36	38	38
**PT18**	2.60 (1.14E-01)	0.60 (6.29E-03)	1.60 (1.96E-01)	1.00 (5.93E-02)	0.27 (1.58E-02)	0.27 (2.80E-02)	0.37 (2.73E-02)	0.40 (4.22E-02)	0.77 (2.10E-02)	1.17 (1.38E-02)	160	75	27	13	29	9	25	13	28	9

TAB S1- Parameters of the model to simulate decision-making in the probability task for the patients (PT), both in ON and OFF

conditions. It can be observed that there is a statistically significant difference in dopamine levels between the two conditions (*** $0.0001 \leq$ p-value ≤ 0.001), proving that the model accurately reflects the difference between the level of treatment given to the patients. No significant differences were found for the other couples of values. However, a significant difference was observed in the final scores between the acquisition and reversal phases in ON and OFF conditions. In Table S1, standard deviations are also reported in parentheses, obtained by applying the minimum percentage variation between 5%, 7%, 9%, 10%, and 20% of the individual parameter, which allows for a non-null covariance matrix.

	DOPAMINE	LEARNING FACTOR	NOISE	LOW SAT	UP SAT	COST FUNCTION	FINAL SCORE		FINAL MODEL SCORE	
							ACQ	REV	ACQ	REV
CS1	1.00 (1.06E-01)	3.50 (5.14E-01)	0.10 (7.57E-03)	0.40 (2.17E-02)	0.75 (1.06E-02)	147	27	27	26	25
CS2	1.00 (3.14E-02)	3.86 (2.74E-01)	0.04 (2.50E-03)	0.38 (7.70E-03)	0.68 (5.00E-03)	25	30	29	30	29
CS3	1.00 (6.23E-02)	0.80 (3.71E-02)	0.02 (2.32E-02)	0.35 (7.32E-02)	0.80 (5.84E-02)	187	40	35	38	35
CS4	1.00 (1.37E-02)	1.13 (2.50E-02)	0.02 (3.59E-03)	0.40 (6.26E-02)	0.95 (6.80E-03)	188	40	16	39	20
CS5	1.00 (1.88E-01)	2.45 (1.24E-01)	0.03 (1.93E-02)	0.28 (2.89E-02)	0.74 (2.30E-02)	135	25	34	28	29
CS6	1.03 (5.58E-02)	2.00 (5.89E-01)	0.07 (1.18E-02)	0.39 (2.77E-02)	0.80 (3.34E-02)	184	34	23	35	26
CS7	1.00 (7.01E-02)	1.55 (1.20E-01)	0.02 (8.67E-03)	0.40 (8.81E-02)	0.93 (1.48E-03)	39	40	29	39	29
CS8	1.00 (8.63E-02)	2.13 (8.56E-02)	0.08 (7.91E-03)	0.40 (1.35E-02)	0.77 (3.85E-02)	285	28	33	32	33
CS9	1.00 (2.40E-02)	1.60 (1.23E-02)	0.06 (2.73E-03)	0.40 (1.86E-02)	0.77 (8.46E-03)	48	33	38	33	37
CS10	1.00 (4.89E-02)	1.53 (8.74E-02)	0.03 (2.39E-03)	0.42 (2.07E-02)	0.65 (2.99E-02)	476	27	30	26	26
CS11	1.00 (2.21E-01)	1.50 (2.42E-01)	0.04 (1.09E-02)	0.40 (6.24E-02)	0.90 (1.00E-01)	183	40	33	38	32
CS12	1.00 (8.07E-02)	1.75 (9.93E-02)	0.05 (1.92E-03)	0.41 (2.75E-02)	0.80 (8.07E-03)	88	33	37	33	35
CS13	1.00 (8.92E-02)	2.2 (1.19E-01)	0.03 (1.10E-03)	0.4 (4.11E-02)	0.93 (2.58E-03)	145	37	33	38	35
CS14	1.00 (4.12E-02)	3.74 (1.02E-01)	0.08 (2.28E-03)	0.40 (5.64E-03)	0.80 (8.43E-03)	31	31	30	29	30

TAB S2- Model parameters used to simulate decision-making in the probability task for control subjects (CS). It can be observed that all control subjects have dopamine values close to 1. None of the parameters show a significant difference compared to the patient parameters in both ON and OFF conditions. In this simulation, the final scores also do not differ significantly between the acquisition and reversal phases, denoting that control subjects are not affected by differences between these two phases.

POWER ANALYSIS:

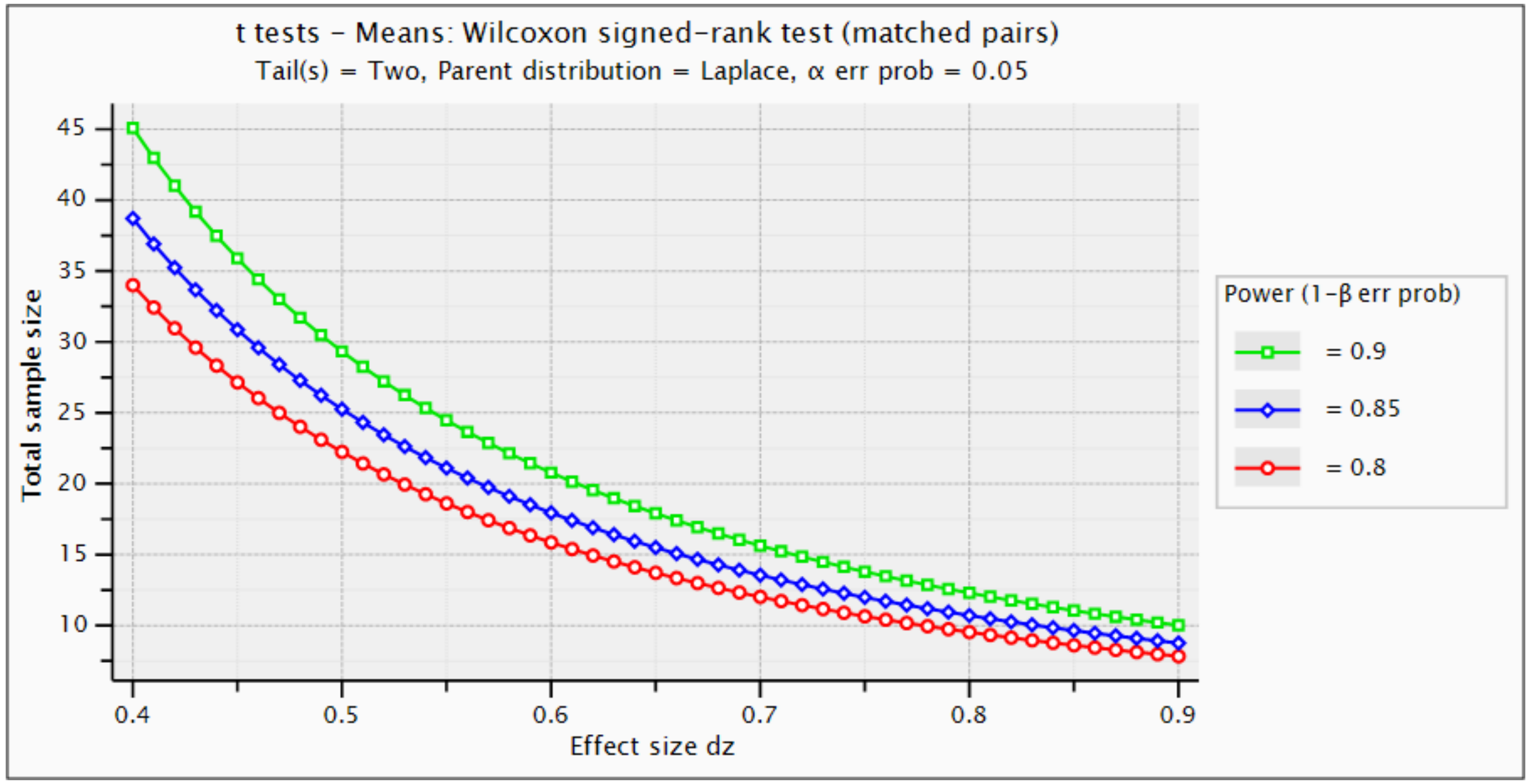

Fig. S3- A priori relationship between sample size and effect size for the comparison of two datasets with non-normal distributions, analyzed using a Wilcoxon signed-rank test for matched pairs. Different curves refer to different powers, with $\alpha = 0.05$.

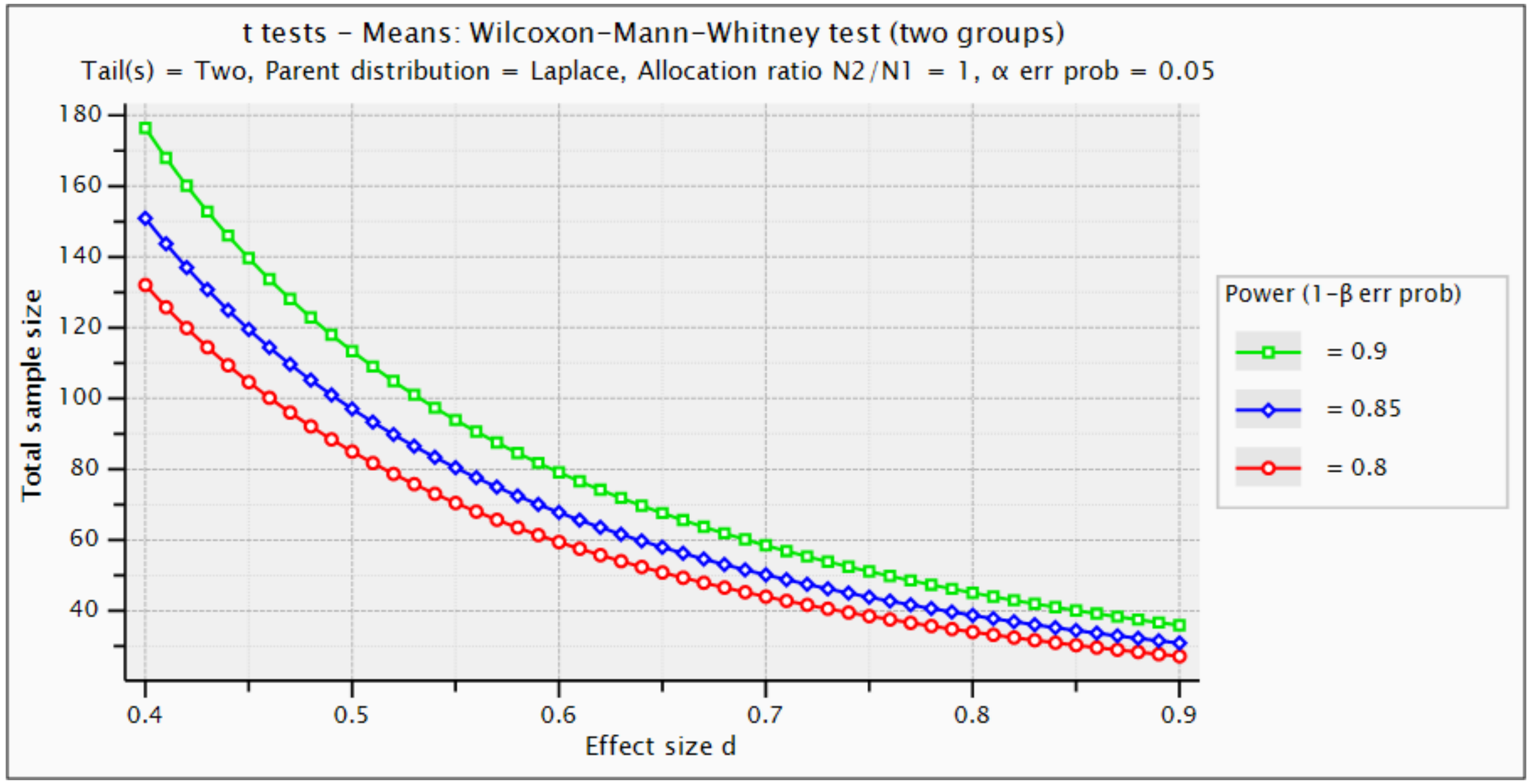

Fig. S4- A priori relationship between sample size and effect size for two independent (unpaired) datasets with the Wilcoxon-Mann-Whitney test. Different curves refer to different powers, with $\alpha = 0.05$. In this analysis, the total number of subjects refers to the sum of participants in the two groups.

	EFF.SIZE	N° SUBJ
CS (ACQ-REV)	0.483	24
PD ON (ACQ-REV)	0.864	9
PD OFF (ACQ-REV)	0.896	8

		EFF.SIZE	N° SUBJ
ACQUISITION	CS-PD ON	0.028	-
	CS-PD OFF	0.439	>100
	PD ON-PD OFF	0.448	28
REVERSAL	CS-PD ON	0.485	92
	CS-PD OFF	0.2419	-
	PD ON-PD OFF	0.173	-

		EFF.SIZE	N° SUBJ
DOPAMINE	CS-PD ON	1.9542	8
	CS-PD OFF	5.5119	4

	**PD ON-PD OFF**	2.7516	3
**LEARNING FACTOR**	**CS-PD ON**	0.3293	-
	**CS-PD OFF**	0.2865	-
	**PD ON-PD OFF**	0.0005	-
**NOISE**	**CS-PD ON**	0.5376	74
	**CS-PD OFF**	0.4175	>100
	**PD ON-PD OFF**	0.1946	-

TAB S3- Effect size computed a posteriori from the available data set (18 PD, 14 CS) regarding the different tests performed during this work. Only when the effect size was larger than 0.4 did we compute the corresponding a posteriori estimated sample size (with power $1 - \beta = 0.8$ and $\alpha = 0.05$). For a two-group test (when we compare CS vs. PD), the sample size is the total number of participants across the two groups (in our case, 18 + 14 = 32).

In all tests that provide a statistically significant result, the sample size is adequate (marked in green). In other cases (marked in yellow), subtle statistically significant differences can be undetected due to an insufficient sample size.

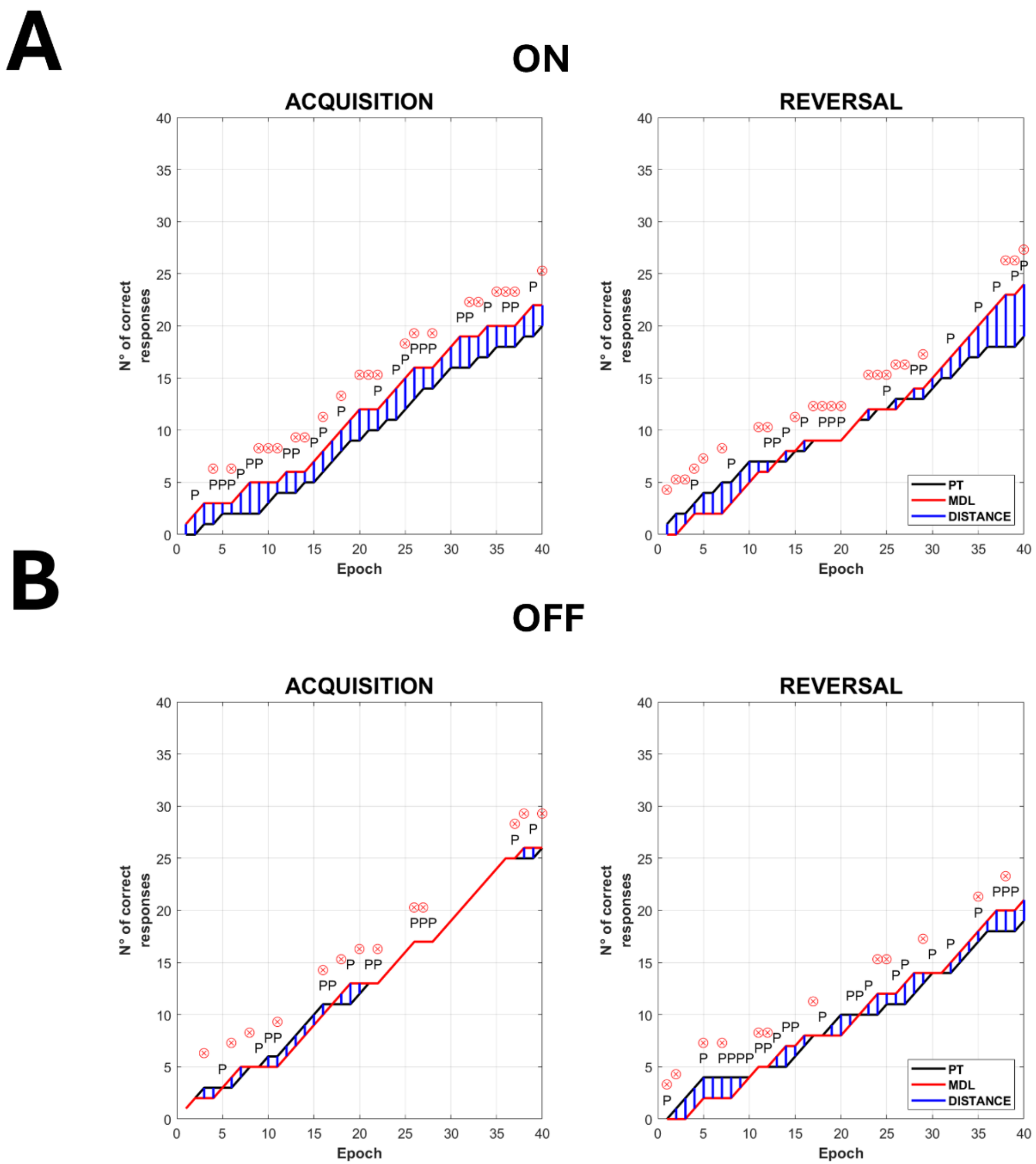
A
ON
ACQUISITION
REVERSAL
N° of correct responses
Epoch
PT
MDL
DISTANCE
B
OFF
ACQUISITION
REVERSAL
N° of correct responses
Epoch
PT
MDL
DISTANCE

Fig. S5 - Model representation of Patient 3 with (Panel A) and without (Panel B) Levodopa treatment. The figure illustrates how the model (red line), incorporating dopamine, learning factor, noise, and lower and upper saturations as inputs, replicates the patient’s behavior (black line) in achieving the final score. The model attempts to reproduce the patient's behavior both in the OFF phase (cost function = 95) and in the ON phase (cost function = 341). This patient is characterized by a very high level of noise input to the model, particularly in the dopamine ON condition, where the patient exhibits a high exploration factor of 0.35 (see Fig. S3).
The parameters for this patient were as follows: in the ON condition — dopamine (1.2), learning factor (3.3), noise (0.36), lower saturation value (0.43), upper saturation value (0.82); and in the OFF condition — dopamine (0.49), learning factor (3.91), noise (0.16), lower saturation value (0.42), upper saturation value (0.83).

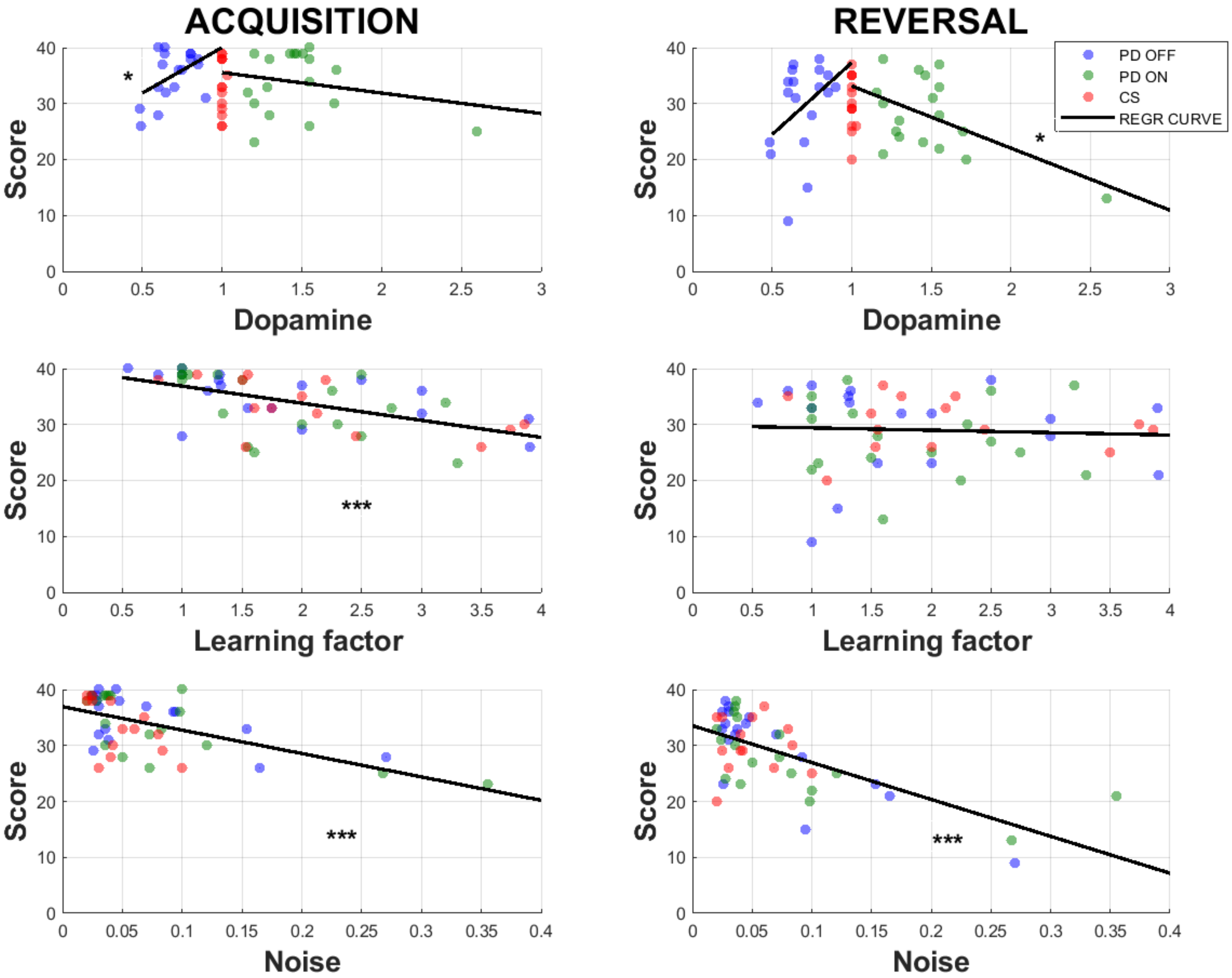

ACQUISITION
REVERSAL
PD OFF
PD ON
CS
REGR CURVE
Score
Dopamine
Learning factor
Noise
*
***

Fig. S6 - Relationship between model parameters and final scores in the acquisition phase (first column) and the reversal phase (second column). The first row highlights a positive relationship between the dopamine levels and final scores, both in acquisition and reversal, for patients without medication (PD OFF). In contrast, a negative relationship is observed for those under levodopa treatment (PD ON). Interestingly, this relationship is statistically significant in the reversal stage. The second row shows that a high gain can negatively impact performance in the acquisition phase. The third row depicts the strong interdependence between high noise values and the learning curve. Statistical results for regression coefficients are shown in the form of asterisks (*: $0.01 \le p \le 0.05$, **: $0.001 \le p \le 0.01$, ***: $p \le 0.001$).
